# Metal-organic framework-based nanomaterials as opto-electrochemical sensors for the detection of antibiotics and hormones: A review

**DOI:** 10.3762/bjnano.14.52

**Published:** 2023-06-01

**Authors:** Akeem Adeyemi Oladipo, Saba Derakhshan Oskouei, Mustafa Gazi

**Affiliations:** 1 Polymeric Materials Research Laboratory, Chemistry Department, Faculty of Arts and Science, Eastern Mediterranean University, TR North Cyprus, Famagusta, via Mersin 10, Türkiye

**Keywords:** antibiotics sensing, endocrinal disorders, fluorescent sensor, hormones sensors, luminescent sensor, MOF nanohybrids

## Abstract

Increasing trace levels of antibiotics and hormones in the environment and food samples are concerning and pose a threat. Opto-electrochemical sensors have received attention due to their low cost, portability, sensitivity, analytical performance, and ease of deployment in the field as compared to conventional expensive technologies that are time-consuming and require experienced professionals. Metal-organic frameworks (MOFs) with variable porosity, active functional sites, and fluorescence capacity are attractive materials for developing opto-electrochemical sensors. Herein, the insights into the capabilities of electrochemical and luminescent MOF sensors for detection and monitoring of antibiotics and hormones from various samples are critically reviewed. The detailed sensing mechanisms and detection limits of MOF sensors are addressed. The challenges, recent advances, and future directions for the development of stable, high-performance MOFs as commercially viable next-generation opto-electrochemical sensor materials for the detection and monitoring of diverse analytes are discussed.

## Introduction

Pharmaceuticals, in particular antibiotics, have become ground-breaking drugs in the medical field for treating a variety of infectious diseases in both humans and livestock. Antibiotics are widely utilized in animal feed to enhance growth and production, which contaminates human food products. Particularly inexpensive quinolones, a class of synthetic antibiotics, are frequently used to treat bacterial infections in animals, particularly fish, cattle, and poultry. However, the presence of these antibiotic residues in foods derived from animals, such as eggs, milk, meat, and fats, can have a number of negative impacts.

Approximately 70 tons of generic and 50 tons of proprietary quinolones are consumed annually in the United States, Japan, South Korea, and the European Union (EU). Recently in China, annual quinolone usage in animal feeds was thought to be in the range of 500 tons, and human consumption was around 1400 tons [1]. Due to their complicated structural makeup, the majority of antibiotics are eliminated unaltered in urine and faeces, which ultimately contaminate natural water sources and soil [2–6]. In environmental samples, antibiotics are currently being found at levels between nanograms and micrograms per litre. Antibiotic abuse and overuse also have serious consequences for the environment and human health. The rise of zombie-like antibiotic-resistant bacteria, which have been linked to severe allergic reactions in people, has been caused in part by the considerable fraction of microbes that have been resistant to a few particular antibiotics [4–7]. Additionally, excessive antibiotic residues in the environment might constitute a major concern by producing conditions including gonorrhoea, tuberculosis, and pneumonia, which complicate their treatment [7–8].

**Figure 1:**
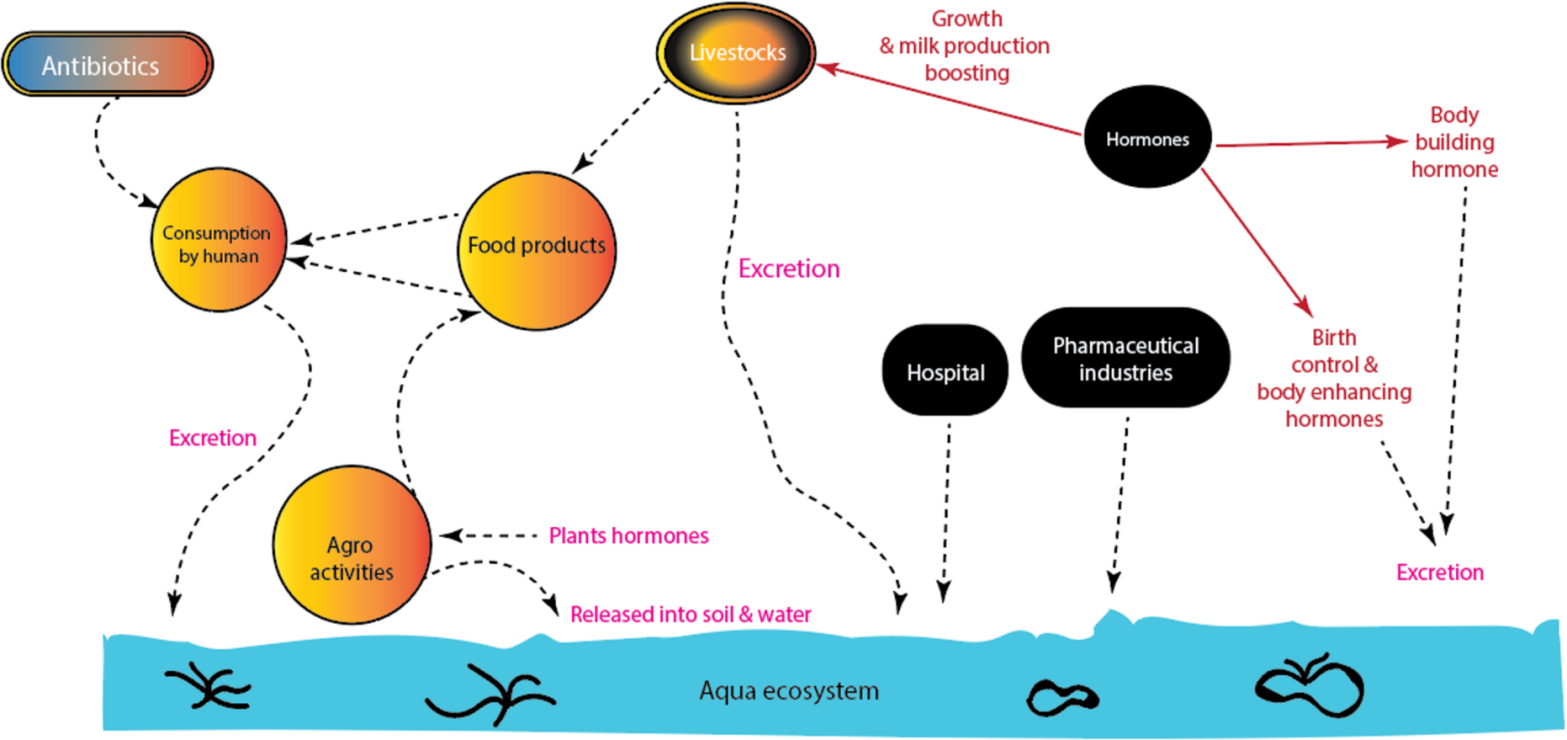
Sources and gateways of antibiotic and hormone residues into environmental media.

Furthermore, the environment is becoming more and more contaminated with both artificial steroids (e.g., 17α-ethinylestradiol, gestodene, trimegestone, antiandrogens, and synthetic estradiol) and natural hormones (e.g., testosterone, progesterone, estradiol, triiodothyronine, thyroxine, and melatonin) [9–11]. Synthetic hormones are often used to accelerate plant and poultry growth, as well as to boost the production of milk in cattle and other animals [9]. In the world today, the use of synthetic hormones for oral contraception, bodybuilding, and weightlifting has increased at an unprecedented rate. These synthetic steroid hormones are endocrine disruptor substances (EDSs) because they have the potential to interfere with physiological functions and negatively impact both the health of the organism and its offspring [12]. Figure 1 shows the several sources and routes by which hormone and antibiotic residues enter the environment.

EDSs are of concern because low quantities can have detrimental effects on the endocrine system and may change an animal’s rate of growth, development, and reproduction [9,12–14]. Even though they are only present in trace amounts in the environment, pharmaceutical substances such as antibiotics and hormones are considered emerging pollutants. Antibiotic overuse and misuse that leads to antimicrobial resistance pose an urgent threat to global public health, killing more than 36,000 people in the United States and being linked to over five million deaths globally in 2019, according to the USA Centers for Disease Control and Prevention report [15].

This is also in line with the World Health Organization’s assertion that “antibiotic resistance” poses a significant financial and societal risk to public health [16]. In fact, according to a 2015 WHO projection, if the current trend in the abuse of antibiotics persists, 300 million people will die prematurely globally over the next 28 years [17]. Considering these environmental and health concerns, a number of regulatory bodies and nations, including the EU, have prohibited the use of chemicals and pharmaceuticals with a hormonal action for the promotion of growth or fattening of livestock through a number of directives due to the endocrine disruptive potential of steroids and natural hormones. Since the COVID-19 pandemic, there have been allegedly higher levels of antibiotics and hormones found in effluents due to increased global antibiotic consumption and the usage of various hormones that promote health [18–19].

**Figure 2:**
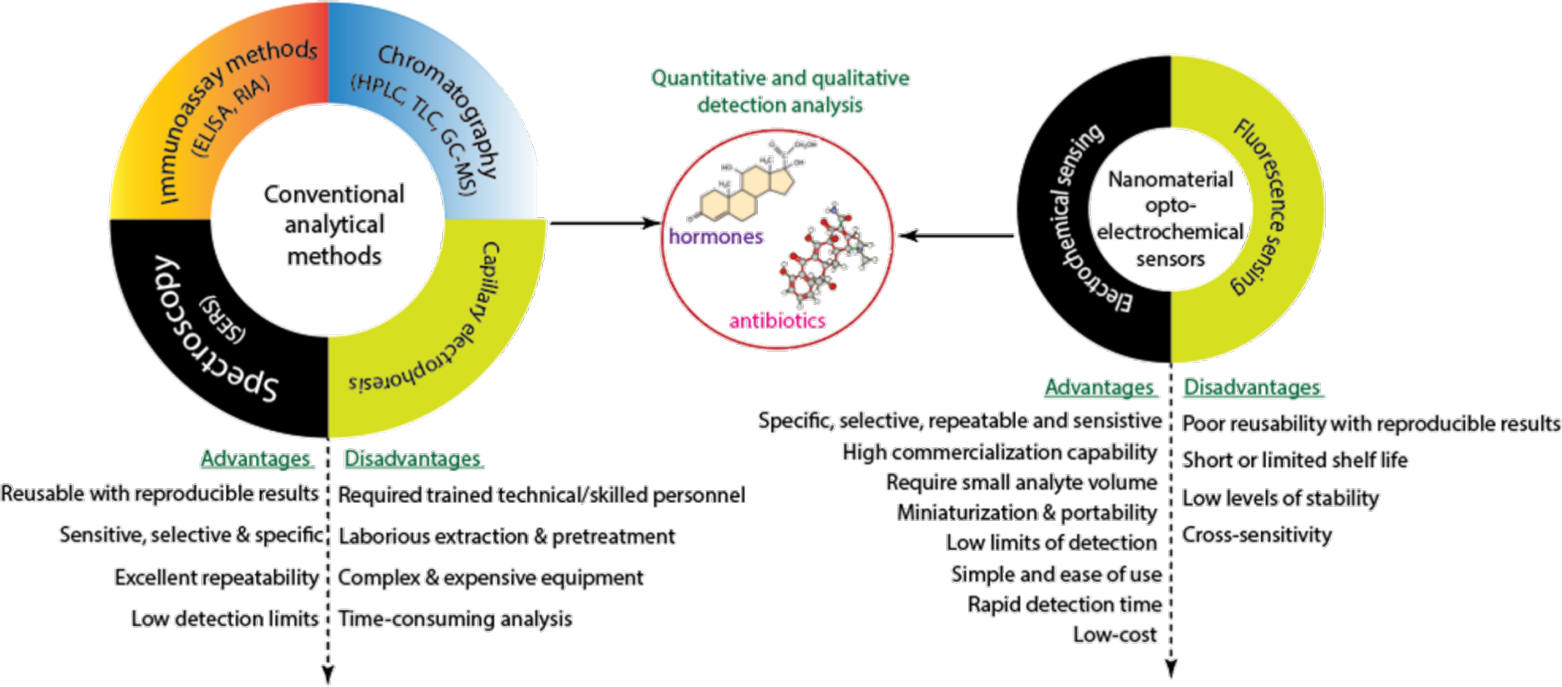
Conventional and portable sensor-based analytical methods for the qualitative or quantitative determination of antibiotics and hormones.

Therefore, research efforts have been concentrated on monitoring and detecting antibiotics and hormones in environmental, clinical, food, and biological samples due to the bioaccumulation, persistence, ecological, and health risks associated with them. However, to detect these emerging contaminants, analytical techniques that are sensitive and selective enough must be developed due to their incredibly low concentrations [2,6–9]. Colourimetry, chromatography, enzyme-linked immunoassay (ELISA), radioimmunoassay (RIA), surface-enhanced Raman spectroscopy (SERS), and capillary electrophoresis are common analytical techniques used to qualitatively or quantitatively determine pharmaceuticals in various matrices because they are sensitive (Figure 2), have a significant tolerable limit of detection (LOD), and are selective in many cases. However, they have a number of shortcomings.

For example, in order to increase the sensitivity of the established immunoassays, Mitchell and Lowe [20] and Wu et al. [21] used the ELISA technique for the analysis of testosterone and progesterone, respectively. Even though this sensitive ELISA approach provided the necessary sensitivity for hormone detection in biological samples, it had a number of drawbacks, including limited specificity, high cost, cross-reactivity, and significant kit variability. The conventional ELISA method necessitates a laborious, multistep separation procedure. A fluorescence polarisation immunoassay, which does not require a separation step, is one development that has been made to get around this restriction [22]. Furthermore, chromatographic techniques such as thin-layer chromatography (TLC), liquid chromatography-coupled mass spectrometry (LC–MS), high-performance liquid chromatography (HPLC), and gas chromatography coupled with mass spectrometry (GC–MS) have demonstrated greater selectivity and lower variability when compared to immunoassays for the measurement of antibiotics and steroid hormones in various complex samples [22–26].

Despite their sensitivity and reproducibility, chromatographic techniques require expensive, bulky equipment, excessive amounts of solvents, extended times for sample preparation and extraction stages, and a variety of stationary phases, making them more difficult to use in labs with limited resources. Additionally, the mobile phase utilized in the separation of antibiotics and hormones affects the chromatography’s ability to detect substances [23]. The advantages of SERS are its rapid detection time, affordable detection cost, and ease of use. It also has a number of drawbacks, such as the necessity for technical experts with the necessary training, the limited selectivity and reusability of substrates, and the degradation of substrates with time, which reduces the signal.

Several portable electrochemical and optical (chemiluminescence, fluorescence, and electrochemiluminescence) sensors have been developed for antibiotic and hormone detection analyses to address the limitations of the standard analytical methods. These sensors’ advantages include high sensitivity, low cost, rapid analysis time, simplified operation without the need for complicated and expensive instrumentation, in situ analyte monitoring, and potential miniaturization. Portability, miniaturisation, and fast signal responses are just a few of the breakthroughs in sensor design made possible by nanomaterials.

**Figure 3:**
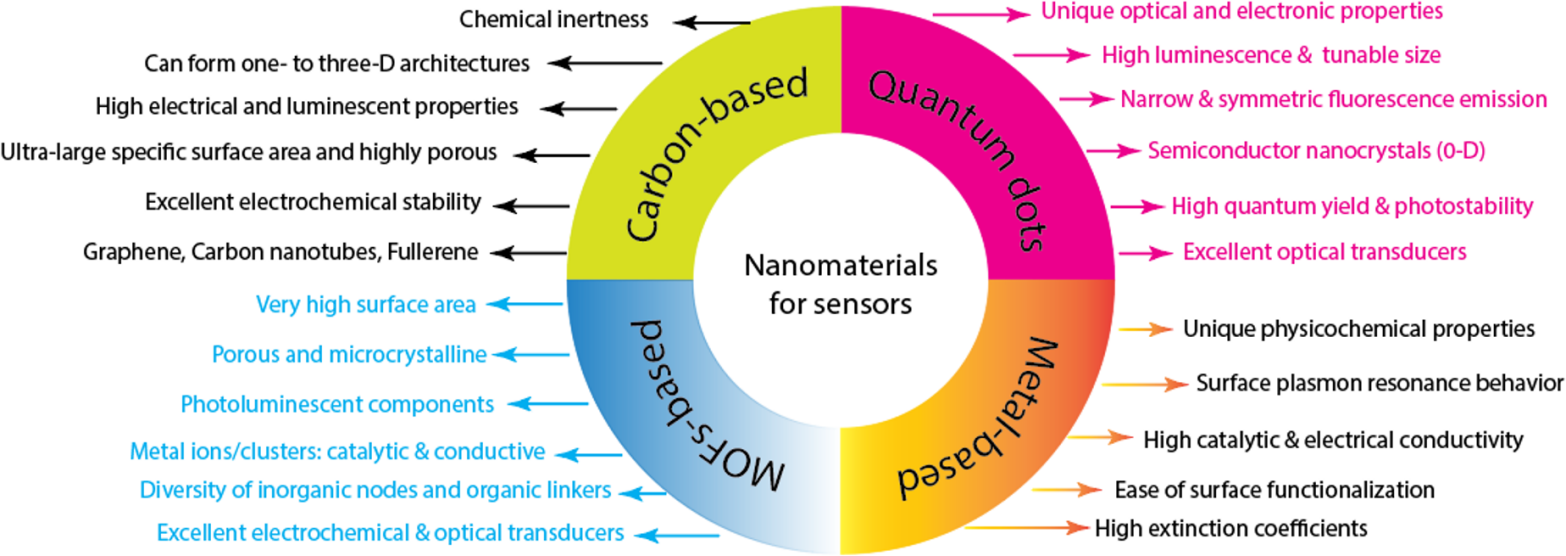
Some desirable features of commonly used nanomaterials for electrochemical and optical sensing platforms.

Nanomaterials are becoming a key component of the analytical procedures required for pharmaceutical, environmental, food safety, and health analyses. They have exceptional physico-chemical and opto-electronic properties, a high surface area-to-volume ratio, and their surfaces are easy to functionalize. Additionally, compared to their bulk counterparts, nanomaterials are particularly sensitive to changes in surface chemistry, enabling nanosensors to achieve extremely low detection limits. Numerous nanomaterials shown in Figure 3 have different functionalities, including high conductivity, good catalytic activity, and optical and plasmonic properties, making them attractive candidates for opto-electrochemical sensing platforms [1,7–10,27–33]. Reliable and sensitive nanomaterial-enabled portable sensors can be quickly deployed to resource-constrained sites to offer rapid and cost-effective monitoring and detection of different analytes without the need for bulky, expensive instrumentation or highly skilled technical experts.

Given the many benefits of nanomaterials, it is anticipated that the incorporation of nanotechnology in sensing would lead to the development of a diagnostic tool for detecting hormone and antibiotic residues in diverse single or multiple matrices. Other researchers have published a number of nanomaterial-based (quantum dots, carbon-based, and metal-based) sensors for the detection of various analytes [1–2,7,9,27–38].

A review paper that offers a comprehensive analysis of current developments based on metal-organic framework (MOF) opto-electrochemical nanosensors for the detection of hormones and antibiotics is still missing, though. This review focuses on a variety of sensing applications that use MOFs as well as the synergistic mechanisms of MOF hybrids or composites that improve sensing performance. It provides a brief summary and discussion of MOF synthesis methods. This review begins by summarising the importance of monitoring and detecting hormones and antibiotics in various matrices. The traditional analytical methods employed for detecting pharmaceutical residues are then briefly described. Comprehensive reviews and discussions were conducted on the mechanisms and performance of opto-electrochemical platforms using MOFs and their hybrids as opto-electrochemical sensors for the efficient detection of hormones and antibiotics in various matrices. Finally, challenges and future perspectives are presented. Therefore, it is anticipated that the concepts presented in this review will stimulate further investigation into MOF-based materials for opto-electrochemical detection of various other analytes (explosives, viruses, and various other emerging contaminants).

## Review

### Opto-electrochemical sensors: mechanisms and challenges

It is crucial to assess the performance of sensors during development using standard metrics such as selectivity, sensitivity, the limit of detection (LOD), and response time. Selectivity refers to a sensor’s capacity to respond to a narrow range of target analytes while resisting interaction with other non-target species; this is often used to determine the accuracy of the results. The LOD of an analyte is the lowest concentration at which it can be consistently detected by an analytical procedure.

**Figure 4:**
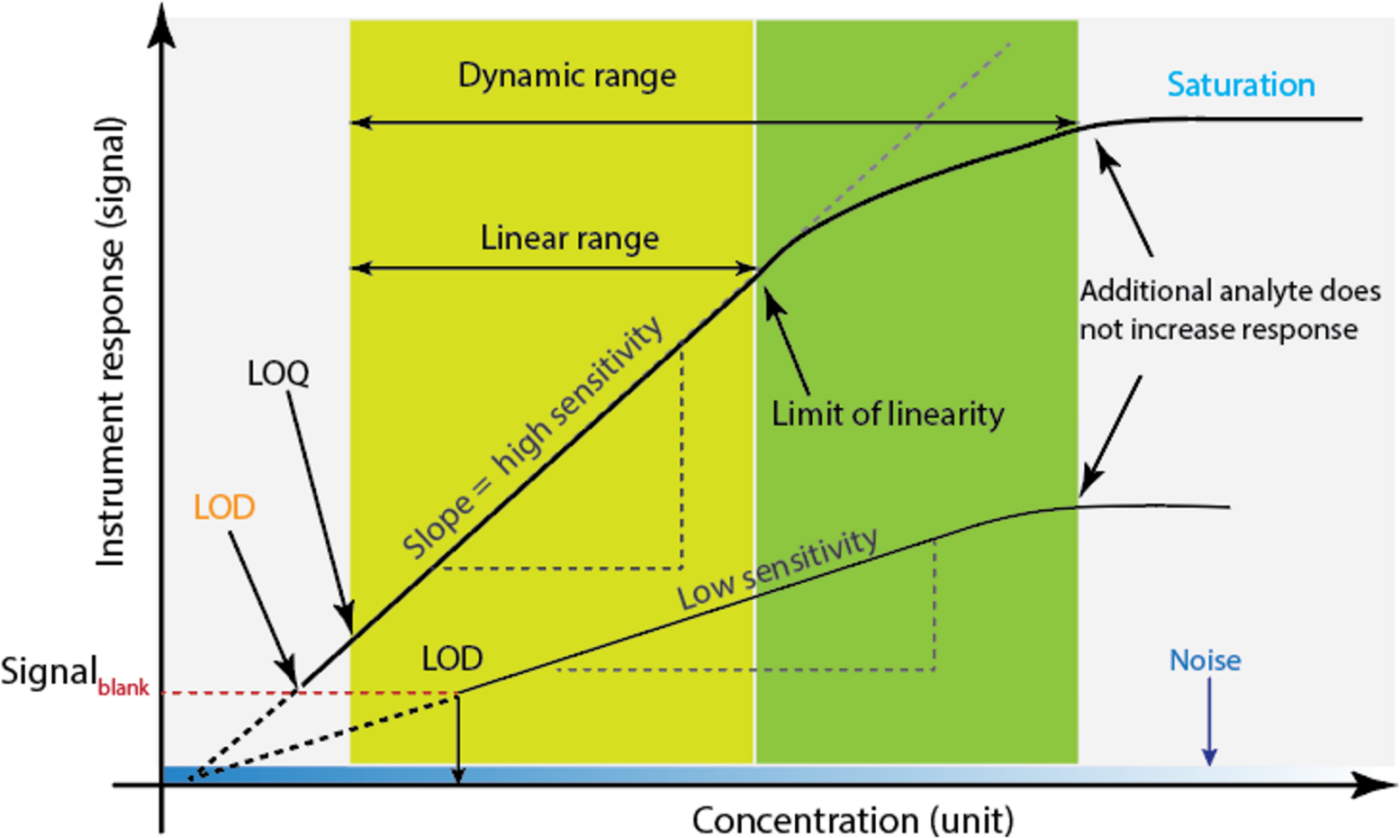
Relationship between sensitivity, the limit of detection, and other key parameters in the sensor.

A measurable signal that can be statistically distinguished from the background or a blank signal must be produced by this concentration (*C*_LOD_) [39]. Sensitivity (*S*) is defined as the ability to change the measured signals (optical or electrical) in response to a change in the amount of analyte. This has a close connection to a sensor’s LOD; the LOD decreases as the sensitivity increases. Generally, it is acknowledged that the *C*_LOD_ can be stated as a function of *S* and *S*_b_ (the standard deviation of a group of blank signals produced via consecutive tests devoid of an analyte): LOD = 3.3·*S*_b_/*S* where *S* = slope of the calibration curve. Also, the slope of the calibration curve can be used to define sensor sensitivity (Figure 4).

The minimum analyte concentration that can be reliably and precisely quantified is expressed by the term “limit of quantification” (LOQ). For estimation, a level of 10·*S*_b_/*S* is recommended. The kinetics of both chemical recognition and signal transduction are correlated with sensor response time. An ideal sensor, when taking into account the aforementioned key parameters, should be specific for the target analytes, sensitive to changes in analyte concentrations, have a rapid response time, have a long lifespan of at least several months, and be small (miniaturised) with the potential for low-cost manufacturing.

### Optical sensing: fluorescent sensors

**Figure 5:**
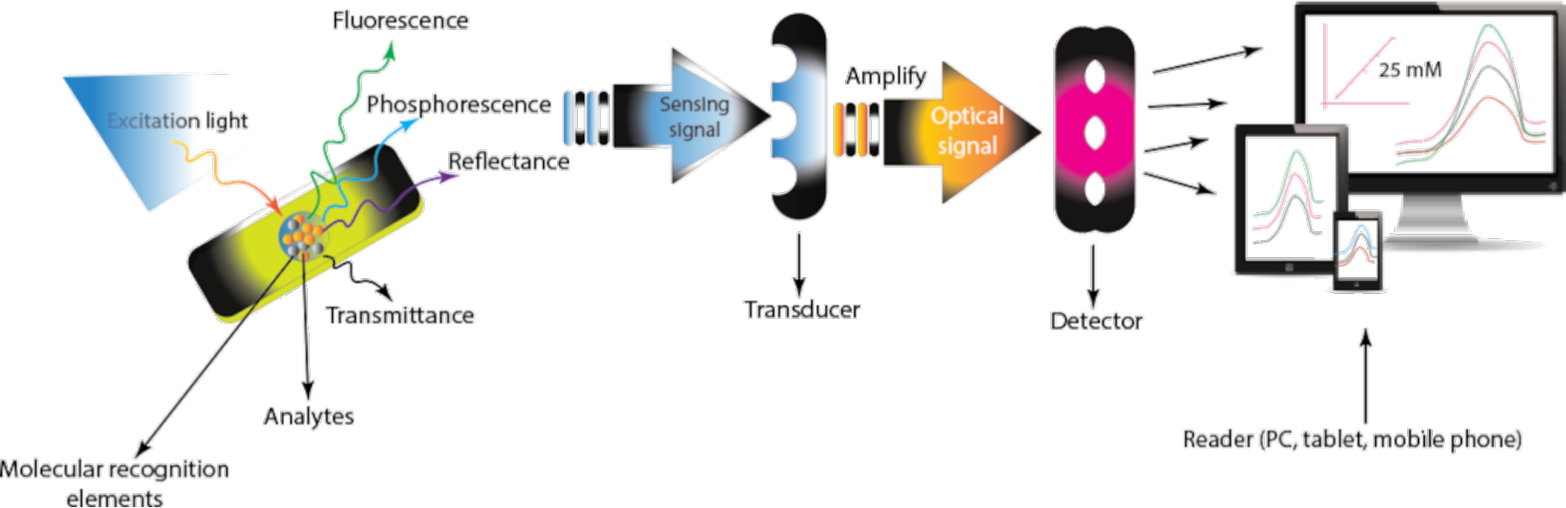
Illustration of optical sensing platforms for analyte detection.

Optical sensors are light-based analytical devices based on the alteration in the measurement of light wavelengths following the interaction of the analyte with the molecular recognition element (Figure 5). Biorecognition elements and signal transducers (chemiluminescence, interferometry, surface plasmon resonance, luminescence, colourimetry, or surface-enhanced Raman spectroscopy), are the key components of an optical sensor. Analyte concentration, existence, and other relevant physical attributes are determined from the optical signals. In recent years, interest in optical methods of hormone and antibiotic detection has grown due to their rapid response times, simplicity of use, and high sensitivity [32–38]. In contrast to other techniques such as colourimetry and surface plasmon resonance, this review will exclusively concentrate on the luminescence (particularly fluorescence) sensing mechanism.

**Figure 6:**
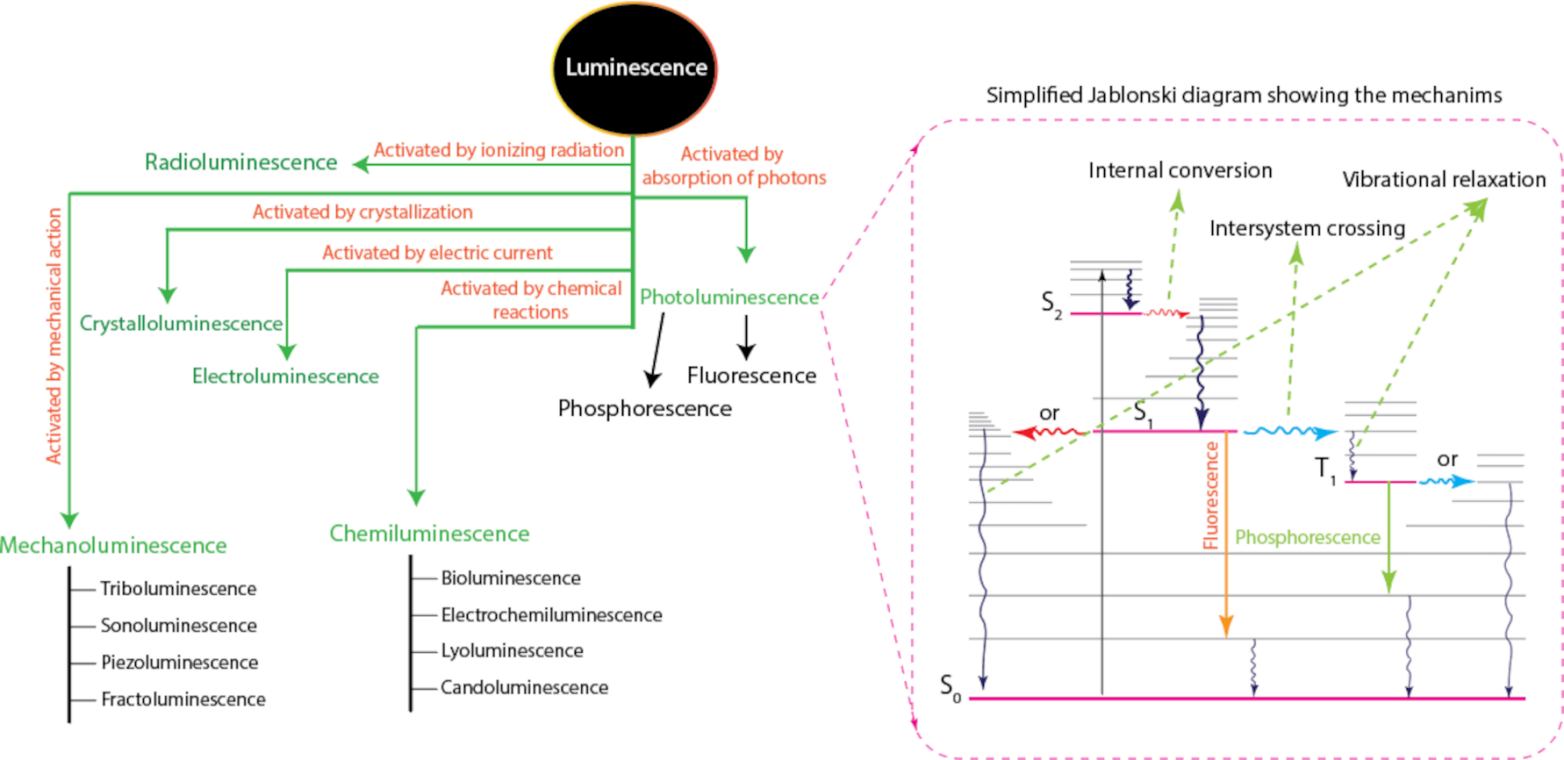
Types of luminescence and mechanisms of fluorescence and phosphorescence.

The basis for optical sensing is the luminescence mechanism, which is the spontaneous emission in the optical range of ultraviolet, visible, or infrared light by a substance without being induced by heat. The emission is caused by electrons transitioning from higher-energy molecular orbitals to lower-energy ones, typically the ground state or the lowest empty molecular orbitals. Luminescence may be caused by intrinsic defects, a particular moiety within the compound (metal or ligand), impurity-induced defects, or it may exist in pure crystals or molecules. According to the manner of the substance excitation, several distinct forms of luminescence are differentiated, as shown in Figure 6. A molecule, nanostructure, or atom must be able to absorb light radiation, resulting in electronic excitation, for photoluminescence to occur, whether it be fluorescence or phosphorescence. The molecule-bound electron in the fluorescence mechanism absorbs a photon and is activated after the analyte interacts with the molecular recognition element. The transition from the ground state (S_0_) to the excited state (S*_n_*, *n* = 1, 2, ...) occurs in femtoseconds [39].

Depending on the wavelength of the absorbed photon, the excited state of the electron may result in the electron occupying any one of a number of possible vibrational levels. In a rapid (nano- to microseconds) transition from the lowest excited state (S_1_) to the ground state (S_0_), the excited electrons relax radiatively through a combination of steps. It is conceivable for the emission to relax to a range of vibrational levels of the S_0_, which gives rise to a bandwidth of potential photon wavelengths. The electron will have lost some of the initial excitation energy through vibrational relaxation, causing the emitted photon to have a longer wavelength and lower energy. Other processes that do not involve light emission can be used to relax the excited state S_1_. These are non-radiative processes that hinder fluorescence emission by competing with it and lowering its effectiveness [39–41]. The shift of the fluorescence spectrum to longer wavelengths with respect to the excitation spectrum is called the Stokes shift.

Fluorescence relaxation processes are all spin-neutral (spin-allowed), and the electron’s spin orientation is always preserved. In contrast, phosphorescence is a separate phenomenon. The electron spin is inverted (spin-forbidden) as a result of a rapid (femto- to microseconds) intersystem crossing from a singlet S_1_ to an energetically advantageous excited triplet T_1_ state. After a long delay (a few milliseconds to a few hundred seconds), relaxation to the singlet S_0_ may take place with the emission of a photon, known as phosphorescence.

Since the majority of these emerging contaminants (antibiotics and hormones) are non-fluorescent, several luminescent or fluorescent materials have been utilised to monitor their levels in different matrices. The choice of the sensor material is crucial to achieving efficient sensing of the target analyte for luminescence-based sensors. Although luminescent sensors have been made using a variety of organic fluorophores and phosphors, the drawbacks of conventional organic dyes for developing luminescent sensors include their toxicity, ease of aggregation, photobleachability, and low capacity for adsorption of the target analyte.

Numerous luminescent materials, including semiconductors, metal complexes, metal-based fluorescent nanoparticles, MOFs, and inorganic phosphors doped with lanthanides, have been thoroughly researched to address these drawbacks. In recent years, theoretical and applied research has focused heavily on luminescent MOFs as an alternative sensing material for fluorescent sensors. These MOFs have an easy-to-functionalize surface, a tunable pore size, intrinsic luminescence, and a desirable adsorption capacity that can enhance MOF–target analyte interactions and transduce these interactions into measurable optical responses. A nanoscale MOF (In-sbdc) with a significant quantum yield of 13% and stable emission in water, for instance, was synthesised by Liu et al. [42] using In^3+^ (metal node) and trans-4,4′-stilbenedicarboxylate (ligand). In-sbdc showed a sensitive response to a variety of tetracycline antibiotics, with detection limits of 0.28–0.30 μM, according to the scientists. Additionally, it was claimed that the antibiotics’ ability to quench In-sbdc was based on an energy transfer mechanism in the sensing system.

**Figure 7:**
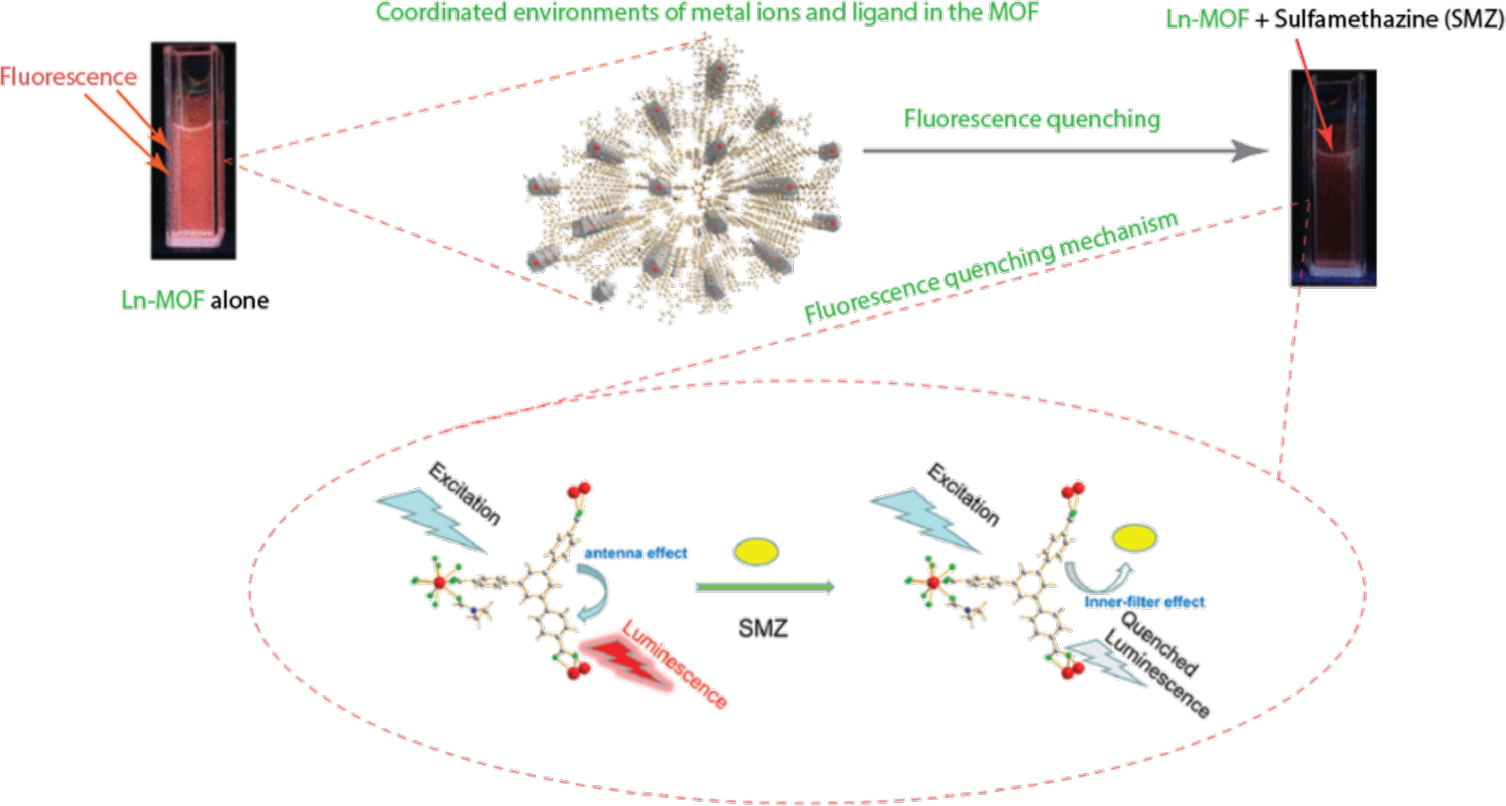
Fluorescent quenching of sulfamethazine by lanthanide-based MOFs. The figure was adapted with permission from [38], Copyright 2019 American Chemical Society.

A water-stable two-dimensional lanthanide-based MOF (Ln-MOF) was synthesised by Ren et al. [38] in a different study to serve as a reversible luminescent sensor for the detection of sulfamethazine (SMZ) antibiotics. According to the authors, the limit of detection for SMZ is 0.655 μM, and the Ln-MOF luminescence is strongly quenched, with a quenching constant of 4.60 × 10^4^ M^−1^. They proposed two potential mechanisms for quenching (inner-filter effect and electron transfer). The overlap between the antibiotic’s absorption spectrum and the Ln-MOF’s excitation/emission spectrum is thought to be the cause of the inner-filter effect, as shown in Figure 7. The second process was linked to an electron transfer from the L-MOF’s conduction band to the antibiotic’s lowest unoccupied molecular orbital. In a related study, Zhang et al. [43] constructed a fluorescent aptasensor to detect the hormone 17-estradiol in urine, water, and milk samples. They were able to detect at a limit of 0.35 nM and determined that the detection process relied on turn-on Förster resonance energy transfer.

The principles of fluorescence quenching (“turn-off”) [37–39] or fluorescence enhancement (“turn-on”) mechanisms [44–45] form the foundation of the majority of luminescent sensors. Static quenching and dynamic quenching are the two basic categories under which luminescence quenching is classified. Dynamic quenching, which is described by the Stern–Volmer equation (Equation 1), results from the interaction and subsequent collision between analyte and fluorophore.


[1] $$\frac{F_{0}}{F}=K_{\text{d}}\left[C\right]+1$$

The luminescence intensities prior to and following the addition of the quencher (analyte), respectively, are represented by *F*_0_ and *F*. The molar concentration of the analyte is [*C*], and *K*_d_ is the Stern–Volmer dynamic quenching constant. The concentration of the quencher will have a linear relationship with the plot of *F*_0_/*F* vs [*C*]. If more than one kind of quenching mechanism, such as dynamic quenching and static quenching, is involved in the process, the plot can shift away from linearity and bend upward or downward. Following that, Equation 1 can be represented as indicated in Equation 2, and by rearrangement, Equation 2 yields Equation 3 as follows:


[2] $$\frac{F_{0}}{F}=\left(K_{\text{d}}\left[C\right]+1\right)\left(K_{\text{s}}\left[C\right]+1\right),$$

[3] $$\frac{F_{0}}{F}=K_{\text{m}}\left[C\right]+1,$$

where


[4] $$K_{\text{m}}=\left(K_{\text{d}}+K_{\text{s}}\right)+K_{\text{d}}K_{\text{s}}\left[C\right]=\left(\frac{F_{0}}{F}-1\right)/\left[C\right].$$

*K*_d_ and *K*_s_ can be determined from slope and intercept of a straight line that is produced by plotting *K*_m_ against [*C*]. In the event that the Stern–Volmer graphs diverge downward, two fluorophore populations are present, but only one of them is accessible to the analyte. Equation 1 is modified in these circumstances to produce Equation 5, where *F* is the total fluorescence and *Fx* and *Fy* are the accessible fluorophore and non-accessible fluorophore fluorescence, respectively. If one fluorophore is accessible to the analyte (*x*) and the other is hidden (*y*), in this case, Equation 6 will give the luminescence.


[5] $$F=F_{x}+F_{y}$$

[6] $$F=\left(\frac{F_{x}}{1+K_{x}\left[C\right]}\right)+F_{y}$$

The fluorophore–analyte complex forms a non-fluorescent ground state, which causes static quenching. The type of quenching mechanism is significantly influenced by the system temperature, concentration of analyte, and viscosity. When the temperature increases, the Stern–Volmer quenching constant (*K*_sv_) values for the dynamic mechanism increase, whereas for the static mechanism, they decrease. This is consistent with the report of Sheta and co-workers [10]. According to the authors, the *K*_sv_ values of the Cu-MOF-based nanosensor were directly correlated with the temperature, indicating that the quenching mechanism between the triiodothyronine hormone and the optical sensor is dynamic. Additionally, the lifetime decay of the fluorophore is unaffected by changes in analyte concentration for static luminescence quenching. The luminescence lifespan in the case of dynamic quenching, however, varies depending on whether the quencher is present or not. The luminescence mechanisms are briefly discussed below, however, the detailed principles of these mechanisms are not covered in this review:

**Fluorescence (Förster) resonance energy transfer (FRET):** In the late 1940s, Theodor Förster put forth the theory, which is based on energy transfer. FRET occurs when an electronically excited fluorophore (donor) transmits its excitation energy to a nearby analyte (acceptor) within 10 nm in a non-radiative manner. In general, the degree of overlap between the fluorophore’s fluorescence emission spectrum and the analyte’s absorption spectrum, the relative orientation of the fluorophore and analyte dipoles, and the distance between them affect the rate of energy transfer [46]. In order to measure the hormone 17β-estradiol in complex sample matrices (milk, urine, or environmental water), Zhang et al. [43] developed a practical FRET-based turn-on fluorescence aptasensor with high selectivity, a low detection limit of 0.35 nM, and an efficient recovery rate of 92.4 to 120.6%. Zhou et al. [47] developed two- and three-dimensional Zn-MOFs with bis-ligand coordination for sensing fluorescent antibiotics (e.g., cefixime, lactams, chloramphenicol, or sulfonamides). The MOFs were found to be efficient, selective, and sensitive toward the antibiotics by their fluorescence quenching behaviour, and it was found that there were distinct overlaps between the analyte’s emission bands and the MOFs’ emission band at 322 nm. The FRET mechanism was therefore thought to be key in detecting these analytes.

**Inner filter effect (IFE):** The IFE was formerly thought of as a fluorescence measurement error. It has gained widespread use in luminescent sensing in recent years as a significant non-irradiation energy conversion technology. IFE is observed when the analyte (acceptor) absorbs the fluorophore’s (donor) excitation or emission light. The IFE-based fluorescence technique is simpler and more versatile than FRET since it does not require covalent bonding between the analyte and the fluorophore or intermolecular interaction [44,48–49]. Although energy transfer occurs in both IFE and FRET, the process can be distinguished by the fluorescence lifetime. In contrast to FRET, where the fluorescence lifetime changes, the fluorescence lifetime in the IFE remains constant both before and after the addition of an analyte to the fluorophore (independent of the fluorescent intensity) [46–51].

**Figure 8:**
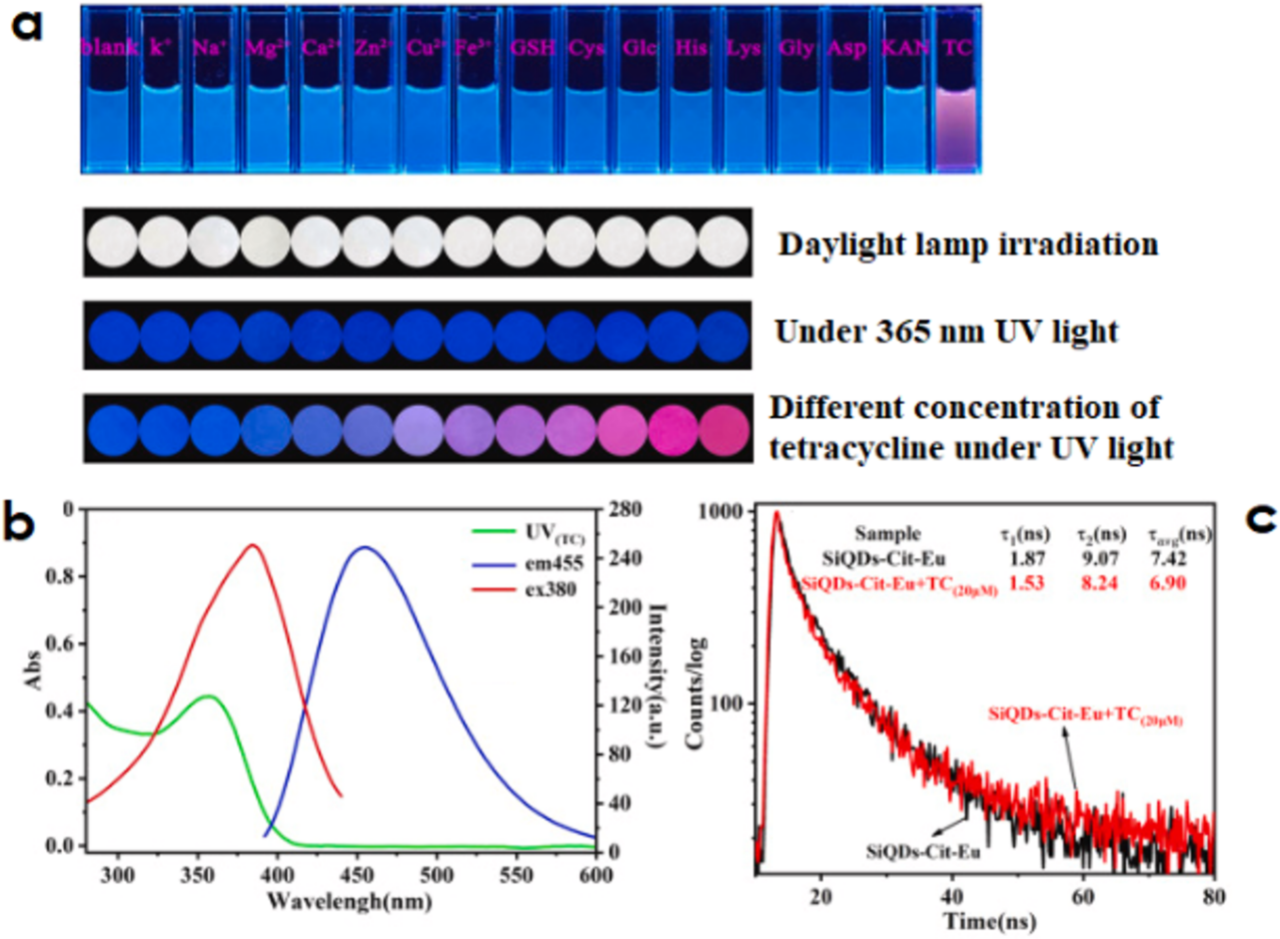
(a) Fluorescence photos for selective responses of an optical nanosensor to tetracycline in the presence of interfering substances. (b) Absorbance and fluorescence intensity of tetracycline and fluorophore. (c) Fluorescence lifetime tests. Reprinted from [52], Journal of Hazardous Materials, Vol. 409, by L. Zhang; Y. Wang; L. Jia; N. Bi, H. Bie, X. Chen; C. Zhang; J. Xu, “Ultrasensitive and visual detection of tetracycline based on dual-recognition units constructed multicolor fluorescent nano-probe“, Article No. 124935; Copyright (2021), with permission from Elsevier. This content is not subject to CC BY 4.0.

The IFE is further categorised into primary and secondary types. The primary IFE is the absorption of the fluorophore’s excitation by the analyte, the secondary IFE is the absorption of the fluorophore’s emission [50]. Yazhini et al. recently developed a highly stable inner filter effect-based Zn-luminescent MOF for the selective detection of tetracycline [48]. They were able to attain a very low detection limit of 0.11 μM and a Stern–Volmer quenching constant value of 1.2 × 10^4^ M^−1^, which demonstrated the accuracy and precision of the MOF’s sensitivity towards the detection of antibiotics. They noticed that the MOF’s (fluorophore) excitation peaks are superimposed over the antibiotic’s (acceptor) UV–visible spectrum. Moreover, the estimated average fluorescence lifetimes before and after the addition of tetracycline were 1.39 and 1.36 ns, respectively, and remained virtually constant. This finding strongly shows that fluorescence quenching is caused by an antibiotic’s internal filter effect and confirms that FRET does not occur. A multicolour fluorescent tetracycline nanosensor (Figure 8) with an ultra-high sensitivity and a detection limit of 7.1 nM in real samples (river and lake water, honey, and milk) was reported by Zhang et al. [52] in another study.

The scientists developed a portable, simple, and affordable optical sensor based on test paper with fluorophore immobilisation for a quick and visible detection of antibiotics (Figure 8a). It was investigated and established how tetracycline-induced quenching occurred. As seen in Figure 8b, the fluorophore’s excitation spectrum and the absorbance band of tetracycline around 357 nm partially overlapped. Moreover, the addition of tetracycline barely affects the fluorescence lifetime of the fluorophore (Figure 8c), which changes from 7.42 to 6.90 ns. These findings confirmed that the steady quenching process based on IFE was responsible for the tetracycline-induced fluorescence quenching.

**Figure 9:**
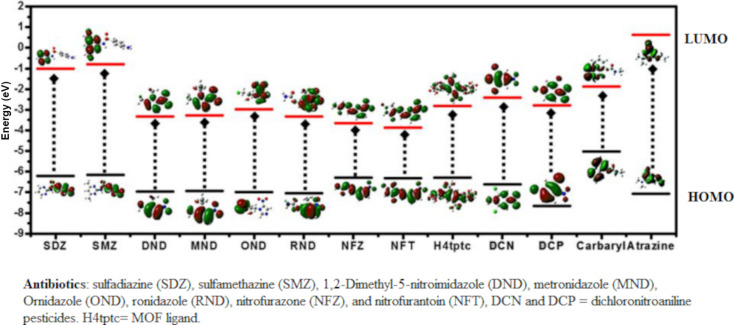
LUMO and HOMO energy levels of the MOF ligand and antibiotics illustrating the PET-induced luminescence quenching mechanism. Adapted from [53], L. Fan et al., “A self-penetrating and chemically stable zinc (ii)-organic framework as multi-responsive chemo-sensor to detect pesticide and antibiotics in water”, Appl. Organomet. Chem., with permission from John Wiley and Sons. Copyright © 2020 John Wiley & Sons, Ltd. This content is not subject to CC BY 4.0.

**Photoinduced electron transfer (PET):** PET is an excitation-induced electron transfer between analytes (electron acceptors) and a fluorophore (an electron donor). Typically, PET results in photoquenching due to an internal redox reaction during the electron deactivation process. For effective quenching, a complex driven by either hydrophobic, van der Waals, or π–π-stacking interactions is generated between the electron donor and the electron acceptor with a separation on a sub-nanometre length scale. The complexation of the electron donor and electron acceptor results in a change in the electron energy levels, which in turn affects the fluorescence signals. Although this complex is capable of returning to the ground state without emitting radiation, exciplex emission is occasionally seen. To detect nitrofuran antibiotics (such as metronidazole, ornidazole, nitrofurantoin, and ronidazole) in water, Fan et al. [53] synthesised a chemically stable zinc-based MOF that functions as a multi-responsive luminescent sensor. With the addition of the antibiotics, the MOF’s luminescence intensity decreased, with LOD values ranging from 19 to 26 ppb. The luminescent MOF-based sensor displayed good anti-interference capability and was highly selective for nitrofuran antibiotics in aqueous solutions. The photoinduced electron transfer was thought to be a key factor in quenching. The electron deficiency of all the antibiotics, which allowed the excited electrons to transfer from the fluorophore’s conduction-band orbital to the analyses’ lowest unoccupied molecular orbitals, was thought to be the cause of the PET-induced quenching. As can be seen in Figure 9, the antibiotics have substantially lower LUMO energy levels than the fluorophore ligands, which accounts for the excellent quenching effects of PET on the luminescence of the MOF sensor [54].

**Photoinduced charge transfer (PCT):** The PCT mechanism relies on the exchange of electrons between acceptor (analyte) and the donor (fluorophore), which results in the alteration of the fluorescence signals. A partial charge transfer of a fully conjugated system occurs in optical PCT sensors. This mechanism involves the complexation of donor and acceptor, which changes the electron energy levels and the fluorescence signals. While PET sensors have the electron donor moiety separated from the fluorophore by a spacer, PCT sensors typically feature an integrated receptor and fluorophore [46].

**Intramolecular charge transfer (ICT):** When the fluorophore contains both electron-withdrawing and electron-donating groups, ICT, an electron transfer process, takes place. In contrast to PET, the electronic states produced by this method are “charge-separated” states. The emission and absorption spectra make it simple to discriminate between PET and ICT. Although there is a significant quenching of luminescence intensity in PET, there is no visible spectral shift. Contrarily, ICT yields environment-dependent changes in luminescence intensity along with sizable modifications in the excitation and emission spectra.

### Electrochemical sensors

Recent years have seen a significant increase in the use of electrochemical sensors [1,7,9,28–33,55–60] that use nanostructured materials as potent analytical tools because of their advantages in terms of portability, affordability, high sensitivity, and ease of fabrication. Through functions such as active large surface area, rapid electrode kinetics, and efficient catalytic activity, the amplification of electrochemical signals based on nanostructured materials has great potential to enhance both the selectivity and sensitivity of electrochemical sensors [55–56].

Even though various sensor systems based on nanomaterials have been published in the literature, it is still difficult to incorporate them into common in situ devices. The most often employed nanomaterials for electrochemical sensors are divided into four categories based on their chemical makeup: (i) metal oxides and metal-based materials (including MOF), (ii) dendrimers and polymer-based, (iii) carbonaceous materials, and (iv) hybrids or composites. Because of their exceptionally high thermal and electrical conductivities, effective catalytic properties, high chemical stability, rapid rate of electron transfer, adequate surface area, and favourable piezoelectric, electronic and gravimetric properties, metal and metal oxide materials with sizes less than 100 nm are the main choice for the design of electrochemical sensors. These characteristics all combine to improve the electrochemical process.

**Figure 10:**
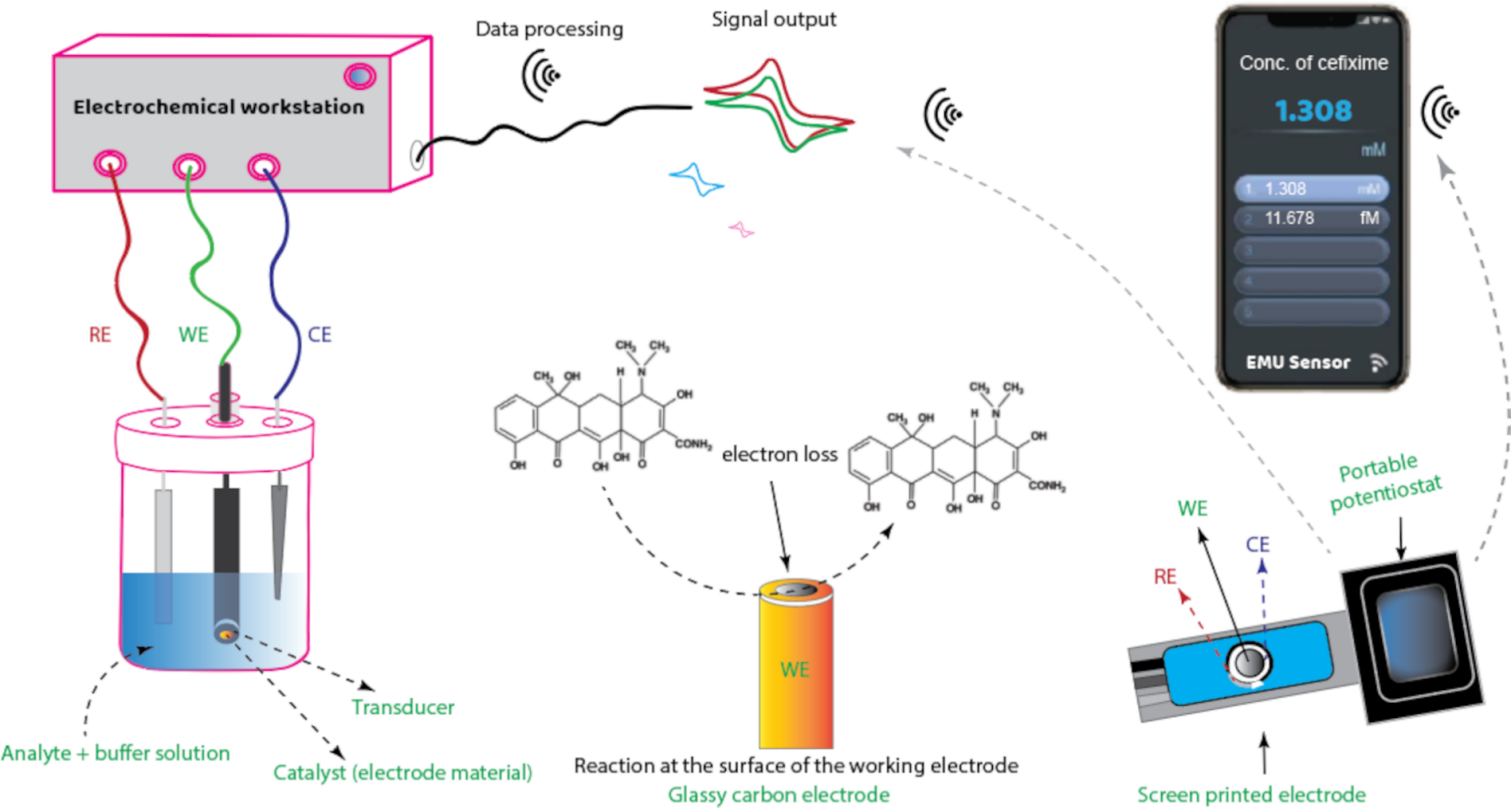
Illustration of electrochemical sensing of an analyte using a standard three-electrode electrochemical cell and screen-printed electrodes with a Bluetooth-enabled potentiostat and mobile phone.

A sensing or working electrode that acts as a transducer, an electrolyte, a diffusion barrier, and a reference counter electrode are the common components of electrochemical sensors (Figure 10).

The target analyte interacts with the recognition layer at the sensing electrode surface to produce an electrical signal that contains the analytical information. Chemical reactions (redox reactions) on the electrode surface are converted by the physicochemical transducer into electrical signals that may be easily identified and shown by electrical equipment. Electrochemical sensor-based techniques can be classified as conductometric, potentiometric, voltammetric, or amperometric, depending on the electrical signal that needs to be measured [56]. Conductivity is measured using conductometric sensors at various frequencies. In potentiometric sensors, a local equilibrium is created at the sensor–analyte interface, and when no current is present, the composition or concentration of the analyte is determined from the potential difference (voltage) between the working and the reference electrode in the form of an electrical signal. The potential of the working electrode is a function of the analyte concentration in the solution. Amperometric sensors use a potential applied between a working and a reference electrode to produce the reduction or oxidation of an electroactive species and then measure the resulting current [61].

In voltammetric sensors that typically have two or three electrodes (the working electrode, the counter electrode, and the reference electrode), the current is measured as a function of the applied voltage at the working electrode. Currently, cyclic voltammetry (CV), differential pulse voltammetry (DPV), square wave voltammetry (SWV), linear sweep voltammetry (LSV), and stripping voltammetry are well-established voltammetric techniques that are frequently used for electrochemical sensing of antibiotic and hormone residues [62]. However, other methods for the identification of these emerging contaminants, such as chronoamperometry and electrochemical impedance spectroscopy, have also attracted a lot of interest [7]. Electrochemical biosensors are created by functionalizing the nanomaterial on the working electrode with biomolecules (e.g., nucleic acids, enzymes, proteins, aptamers, and immunoglobulins) to realise an analyte-specific reaction. Because of their modification with biomolecules, some electrochemical biosensors have shown higher specificity and selectivity than unmodified electrochemical sensors; nevertheless, they have shorter lifetimes and poorer levels of stability due to the rapid degradation of the biomolecules.

Extensive research has been focused on the development of different high-performance electrode materials via modification of the materials’ surface with functional groups or doping with various metals or via the formation of nanocomposites. Because of their large surface area, ordered structure, tunable physico-chemical properties, and good absorbability, MOFs exhibit high potential among the various materials used for electrochemical sensor electrodes [63].

Strong interactions between the functional groups of MOFs and the target biomolecules via electrostatic forces, stacking, and/or hydrogen bonding, which lead to high accumulation of the target analyte, are another factor that supports the development of electrochemical sensors. However, because of the high proportion of organic ligands, most MOFs have poor electrical conductivity, which lowers their electrochemical detection performance [64]. Researchers have focused on various research efforts to improve the conductivity and amplify the electrical signals of MOFs by combining them with other highly conductive materials (such as carbon materials, metal nanoparticles, or metal oxides) [63–69]. This is motivated by their large surface area, which can facilitate the loading of nanoparticles. Additionally, MOFs have been converted into their electrochemically active derivatives, such as mesoporous carbon composites and porous metal oxides, to achieve an improved electrochemical performance [63,67].

In a recent study, Tang et al. [29] used an ultrasonication and reduction process to combine nanostructured Ag nanoparticles with a MOF (ZIF-67) to fabricate nanopinna-based composite electrochemical sensors for acetaminophen detection. The results showed a wide linear detection range with a detection limit of 0.05 μM. Due to the synergistic effects of the wide porosity and high specific surface area of the MOF and the outstanding catalytic activity and high conductivity of Ag nanoparticles, the hybridisation improved the electrochemical performance of the sensor. A novel electrochemical aptasensor based on Fe MOFs was reported by Song and co-workers [70]. In order to increase the sensor’s sensitivity to the detection of antibiotics, the MOF was further modified using carbon nanofibres and gold nanoparticles using a variety of techniques. In the presence of seven other antibiotics, the electrochemical sensor showed good selectivity and stability, with the lowest detection limit of 0.01 nM. Tetracycline concentrations in samples of lake and tap water were precisely quantified by the sensor when its performance was tested using real samples. An electrochemical sensor for the detection of tetracycline based on a glassy carbon electrode modified with MIL-53 (Fe) was published by Chen and co-workers [71]. The authors claim that the electrochemical sensor has a good linear relationship for tetracycline detection in the range of 0.0643–1.53 mol L^−1^ and that it has a high anti-interference ability.

It is important to note that a number of MOF-based materials have been used as opto-electrochemical sensors for the dual detection of hormones and antibiotics using hybrid optical and electrochemical methods. As two non-interfering and mutually independent signals are obtained, the likelihood of inaccurate results is reduced. For example, Rani et al. [50] synthesised a gold nanoparticle-decorated amine-functionalized Zr-MOF through a solvothermal synthesis process in order to detect nitrofurazone antibiotics via dual-mode (opto-electrochemical) sensing. Utilizing both the fluorescence of the Zr-MOF and the electrochemical properties of nitrofurazone antibiotics, a more accurate signal and diversified results were obtained. With a LOD of 3 × 10^−9^ mol·L^−1^ in the electrochemical mode and 5.5 × 10^−9^ mol·L^−1^ in fluorescence sensing, the prepared sensor demonstrated a wide linearity range for the detection of the antibiotic.

### Metal-organic frameworks: properties and synthesis

**Figure 11:**
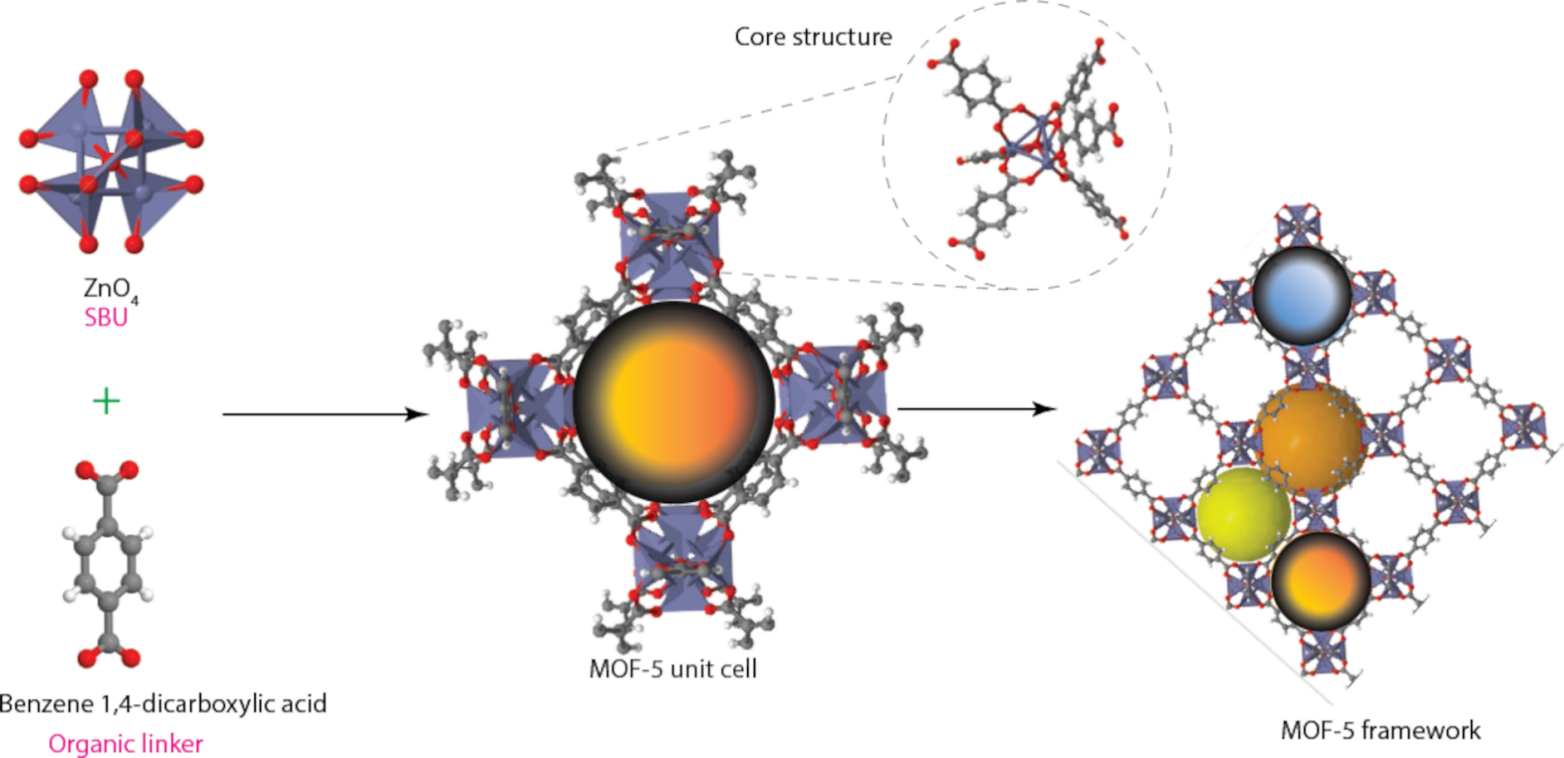
MOF-5-unit cell and framework synthesised from ZnO_4_ secondary building units (SBU) and organic linker. The accessible volume in the pore is depicted in the form of spheres. Images were drawn using Illustrator and Diamond software-based crystallographic data.

In recent years, there has been a lot of interest in metal-organic frameworks (MOFs) [72–75], which are extremely porous materials. They are highly crystalline materials with extremely low densities (0.1–1 g·cm^−3^), the highest specific surface areas (165–7800 m^2^·g^−1^) currently known, tunable surface properties, good mechanical and thermal stability, intriguing and controllable morphologies, and uniform yet tunable pores that are created by the self-assembly of metal ions or clusters and organic ligands (i.e., “linkers” or “struts”) through coordination bonding [30,67,76–78]. The development of the first MOF and covalent organic framework (COF) is credited to Omar Yaghi of Berkeley University of California. In particular, Yaghi reported in 1995 on the synthesis and crystallisation of the first MOF in which metal ions are connected by carboxylate linkers (ligands) [67], as shown in Figure 11.

The metal ions act as nodes, connecting the ligands’ arms to create a repetitive, cage-like structure. The internal surface area of MOFs is incredibly vast because of their hollow structure. However, the chemical stability of the older MOFs was not very good, especially those that contained pure tetrahedral divalent metal units.

However, an incredibly stable and highly functional MOF was published in 1999 by Yaghi and co-workers [79], and several other MOFs have been synthesized and reported by various other researchers since then. MOFs are also referred to as “coordination polymers”. Secondary building units (SBU), which determine the final topology and hence the properties of the MOF framework, are coordination complexes generated between the donor atoms of the ligands and the metal ions. By regulating how many ligands can bind to the metal and in which direction, the coordination preference of the metal, for instance, affects the stability, size, and nature of the formed pores.

### Electronic and optical properties of MOFs

In addition to the physicochemical characteristics of MOFs already described, the following is a brief outline of some of the desirable qualities of MOFs that are required for developing opto-electrochemical sensors.

**Electronic properties:** Electrostatic potential, density of states, electron density, bandgap, and conductivity are some of a MOF’s crucial electrical properties. Because of the typically insulating properties of the organic linkers and low levels of π–π-orbital conjugation, significant electrical conductivity is uncommon in MOFs. Even though MOFs’ electronic characteristics have not received much attention, their potential as electrically conductive porous materials has only recently come to light. Recent research has demonstrated that the nature of metal clusters, their size, and the kind of organic linkers all affect the MOFs’ electronic properties [80–81]. To clarify the electrical properties of MOFs, Kuc et al. [80] used tight-binding simulations based on density functional theory with periodic boundary conditions. In comparison to the building units, they noticed that MOFs have a charge distribution that remains constant, and their electronic properties show a wide range of bandgap energies categorized as insulators or semiconductors. The authors pointed out that metal clusters (for example, isoreticular MOFs) essentially define the overall electronic properties of MOFs and provide MOFs with the characteristics of a wide-bandgap semiconductor like ZnO. The size of the organic linker and the hybridization of the central atom of the linker both affect the bandgap values of MOFs, which range from 1.0 to 5.5 eV. Organic linkers with more conjugated carbon atoms tend to have smaller bandgaps. Additionally, the bandgap narrows as the number of sp^2^-hybridised carbon atoms in the linker increases because more π states contribute to the overall band structure. Additionally, computational analyses have shown that the functionality of the linker, such as nitro, carboxylic, or amine, can affect the electrical characteristics of MOFs, particularly the bandgap. The p orbital interactions of the functional group with aromatic carbon atoms, which might produce localization of donor states close to the aromatic ring, were suggested as the source of the bandgap modulation due to changes in the functionality of the organic linker [82]. Due to MOFs’ low resistivity and rapid charge carrier mobility, some researchers [80–83] have recently suggested that MOFs occasionally exhibit superconductive behaviour. The presence of metallic state bands, which correspond to π-type crystal orbitals centred on ligand atoms and metal ions, as well as the availability of the most extensive mobility pathways between the sheets, clarified the conductive behaviour even further. Through variation in temperature resistivity, Clough et al. [83] demonstrated band-like metallic conductivity in cobalt-based MOF. The ferroelectric characteristics of MOFs have not yet been extensively investigated experimentally. However, calculations based on density functional theory have been used to examine aspects of MOFs, including ferroelectric polarisation. Particularly appealing multiferroic and ferroelectric properties have been associated with MOFs containing formate ligands [84]. The incorporation of guest molecules (dopants) into the porous network of MOFs, the use of a distinct linker, and the creation of MOF composites with other conducting materials (carbon-based, metal, and mixed metal oxide nanoparticles) are just a few experimental strategies that have been suggested to further improve the electronic properties of MOFs.

**Figure 12:**
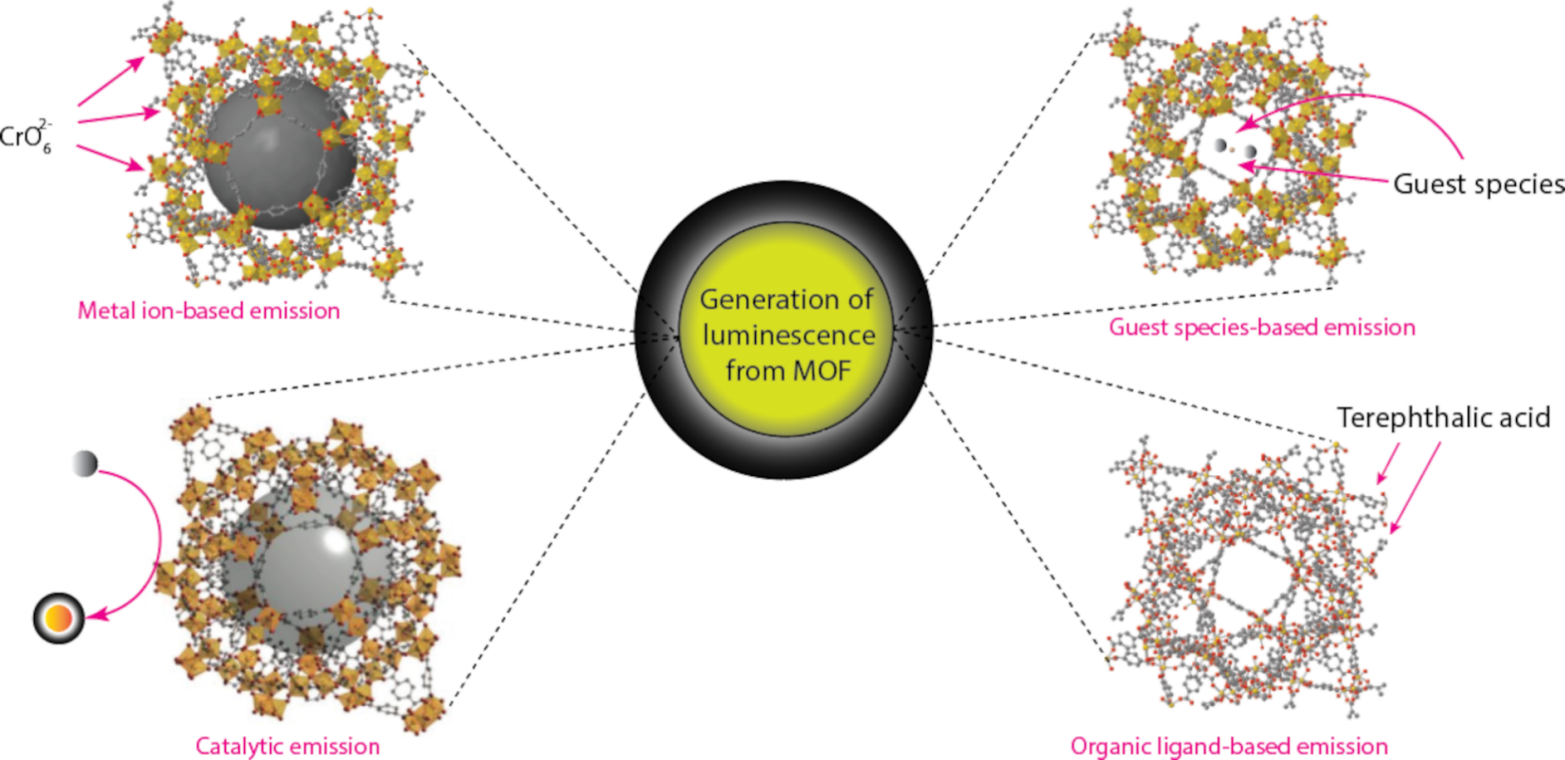
Methods frequently used to produce luminescence from MOFs.

**Luminescence properties:** The fact that MOFs exhibit not just fluorescence but also phosphorescence and scintillation has drawn attention to their optical capabilities for some time. Due to their hybrid composition, MOF materials are capable of a variety of emission phenomena (Figure 12) that are uncommon in other material classes. For example, MOFs show metal-based emission (typically the lanthanides) and antennae effects, exciplex and excimer emission, analyte-based sensitization and emission, and ligand-based luminescence [85]. The MOF structure, which determines the bonding geometry, the metal electronic configuration, and the accessible states of the metal ions in the framework versus the HOMO–LUMO gap of the organic ligand(s), all have an impact on the emission that results from MOFs.

*Metal-based luminescence:* The incorporation of lanthanide elements into the MOF structure results in the most frequent occurrence of metal-based luminescence [85–87]. This process is known as ligand-to-metal charge transfer (LMCT). The lanthanide series consists of fifteen elements, ranging in atomic number from 57 for lanthanum to 71 for lutetium. The chemically related elements scandium and yttrium are also included in this group of fifteen, which are commonly referred to as rare earth elements. All lanthanoids, with the exception of Eu^2+^ and Ce^4+^, exist in the trivalent oxidation state (Ln^3+^). Ln^3+^ has a firmly seated 4f orbital in its ground state electronic configuration ([Xe]4f*^n^*, where *n* = 0–14), and the electrons in this orbital are protected by a wider radial expansion of its filled 5s^2^ and 5p^6^ subshells, which create 4f orbitals as inner orbitals. As a result, the spin–orbit interaction, which is the basis for their intriguing photophysical features, dominates the extremely minor and less significant crystal field influence on the deeply seated 4f orbitals. For lanthanoids, three different forms of electronic transitions are possible, namely the 4f→5d transition, broad charge transfer transitions, which are intense but broad, and sharp narrow bands (4f→4f transitions), which are very weak in intensity. Compared to the 4f→5d and the charge transfer transitions, which are Laporte-allowed transitions (high probability of occurrence), the 4f→4f transitions are Laporte-prohibited transitions (low probability of occurrence). However, due to parity selection principles, the direct excitation of lanthanoids into excited 4f states is practically limited, suppressing the lanthanoid’s sharp luminescence and leading to poor quantum yields and weak absorbance. Strong vibronic coupling between the desired lanthanoid and a highly absorbing ligand is a popular method of resolving this issue since it allows for direct energy transfer from the more accessible excited states of the ligand to the suitable metal energy level. The “antenna effect”, which is the name given to this coupling, causes a significant rise in luminescence [85]. Particularly, the lanthanide ion emits its distinctive luminescence as a result of being indirectly activated by energy or electron transfer through its surrounding ligands. A major benefit of this method is its significant Stokes shift, which makes it easier to distinguish between lanthanide luminescence and its excitation light and ligand-centred luminescence. Second, it significantly increases the luminescence quantum yield at room temperature conditions [87–88]. Although nearly all lanthanide elements display photoluminescent capabilities, only two lanthanoid ions, Eu^3+^ and Tb^3+^, have intense visible luminescence in red and green, respectively. These two ions are the ones that are most frequently used when synthesising MOFs for optical sensing applications. Note that phosphorescence, which is typically relatively weak at ambient temperature due to solvent quenching and self-quenching of the long-lived excited state, constitutes the majority of lanthanide luminescence. The possibility of solvent quenching and self-quenching, however, has been virtually eliminated in MOFs since lanthanide ions are entrenched in the network of organic ligands. So strong is the luminescence produced by lanthanides in MOFs that it can even be seen with the naked eye. Also, actinide-containing MOFs exhibit luminescent behaviour [86]. The metal ion can have different levels of impact on the emission depending on the electronic structure of the metal and the relative energy of the metal and linker orbitals. To develop luminescent MOFs, a variety of transition metals have also been combined with different ligands. Note that the linker, not the metal, is often the focal point of luminescence from such MOFs [85]. Because ligand–metal transitions (d→d) may strongly reabsorb or quench the fluorescence produced by the organic linker via energy or electron transfer through the partially filled d orbitals, the emission from transition metals with unpaired electrons is often not strong. But MOFs containing transition-metal ions devoid of unpaired electrons, particularly those with d^10^ configurations, can produce remarkable linker-based luminescence [85–87].

*Linker-based luminescence:* In MOF chemistry, a variety of organic linkers have been used, many of which contain rigid backbones that have been substituted with multiple carboxylate groups for metal coordination. As linkers in MOFs, organic compounds with fused π rings and strong conjugation are frequently used. Due to their stiffness, they are frequently both very emissive and intensely absorbing, which can result in a variety of intriguing luminescence features in the material. In MOFs, ligand-localised emission, ligand-to-metal charge transfer, and metal-to-ligand charge transfer are among the typical mechanisms of linker-based luminescence. Terephthalic acid, trimesic acid, 5-sulfosalicylic acid, 4-aminobenzoic acid, and nitrilotriacetic acid are a few of the commonly used linkers. Note that the scope of this review does not include a thorough discussion of the linker types. Depending on the type of analyte and the composition of the MOF, luminescence intensity in MOFs can either be quenched or enhanced.

Due to their exceptional characteristics, MOFs have found usage in a variety of fields, including sensors, gas adsorption, energy storage, drug delivery, catalysis, water treatment, and bio-medical imaging [89–101]. Numerous early MOFs produced from divalent metals displayed excellent porosity but were inappropriate for usage in water, moisture, or acidic or basic environments due to stability problems, which significantly restricted their further use and commercialization. When exposed to atmospheric moisture, MOF-5, for instance, eventually degrades [95]. As a result, in recent years, there has been an increase in interest in the chemical stability of MOFs. The stability of MOFs in various environments has begun to be addressed, and researchers are working to create more robust framework structures. The stability of MOFs is thought to be a result of the comparatively brittle coordination bridges that sustain the framework structures. For example, carboxylate-based ligands are considered hard bases and are capable of forming stable MOFs with high-valence metal ions (Cr^3+^, Fe^3+^, Al^3+^, Zr^4+^, and Ti^4+^), resulting in a more stable framework. Because of their exceptional stability in water and even in acidic or basic environments, high-valence metal-based MOFs like the MIL (Material Institut Lavoisier) series, including MIL-53 (synthesised in 2002), and the Zr^4+^-based MOFs have drawn a lot of attention. This trend has continued in recent years, with an increasing number of stable MOFs being synthesised and reported. Due to the development of more stable MOFs, the sensing of different analytes from industrial, food, water, clinical, and environmental samples is now included in the recent expansion of MOF-based material applications.

Because of their high sensitivity, extensive porosity, controllable architectures, facile functionalization, large surface area, and low detection limit, MOF-based sensors have received a lot of interest. Additionally, MOFs are a perfect choice for opto-electrochemical sensors because of their stability and the variety of interactions that are made possible by the inclusion of both inorganic and organic moieties in the structure [28,34–37,50,67]. For the development of optical sensors, MOFs are enormously beneficial since their organic ligands with conjugated or aromatic moieties can generate fluorescence when exposed to radiation. Also, the metal ions (such as inorganic clusters and lanthanides) can produce photoluminescence [34–37].

The uses of MOFs in electrochemical sensing have only been lightly investigated because the majority of MOFs exhibit insulating properties. The potential of MOFs as electrochemical sensing platforms has been shown in a number of recent studies. The following factors contribute to MOFs’ electrochemical sensing abilities: (i) They have vast surface areas, highly active catalytic capacities, and unsaturated metal coordination sites, which make them ideal materials for coating electrodes used in sensing applications. (ii) MOFs benefit from the mass transfer of the analytes due to their high porosity, which can effectively amplify the electrical signals and increase detection sensitivity. (iii) Through the use of size exclusion effects, MOF-based matrices can exhibit good selectivity towards particular analytes due to the tunable shape and size of the accessible cavities and channels within the framework [67,76]. MOFs thus provide intriguing and noteworthy benefits over other materials, considering their electronic and luminescent properties.

Electrochemical sensors display good stability and rapid response over a suitable humidity range. However, under extreme conditions, the electrochemical sensor lifetime is influenced by temperature and humidity. Because of the structural features of MOFs, the electrochemical sensing capabilities of MOFs are not considerably impacted by humidity. By exposing more active sites during the interaction with water molecules, MOFs’ increased porosity and larger specific surface area could limit the effects of humid environments. In practical settings, the key factor contributing to the decreased resistance of the majority of MOF-based sensors under high humidity is an increase in their conductivity. Increased humidity causes the adsorbed water molecules to form an interconnected water film on the MOF surface through hydrogen bonding, which promotes proton conduction and raises the sensing materials’ conductivity. Therefore, MOFs are capable to exhibit significant conductivity changes under various humidity environments by ligand choice and structural modification.

The inclusion of hydrophilic ligands and metal oxides into MOFs has been the focus of study to further increase the stability of MOFs under humid environments. For instance, metal–oxygen groups such as Ti–O and functional amino groups (NH_2_) are hydrophilic, and MOFs based on these hydrophilic groups are anticipated to exhibit a decrease in the electrochemical impedance when the humidity levels rise. Additionally, Cu-based MOFs with high hydrophilicity resulting from the existence of uncoordinated active sites are helpful in the preparation of stable electrochemical sensors that can function well in extremely humid environments. Al-based MOFs, such as CAU-10 and MIL-96, are renowned for their great stability and strong hydrophilicity and are consequently anticipated to function well in humid environments.

### Drawbacks of MOFs as opto-electrochemical sensors and recent advances

The classic MOFs have some limitations when utilized as opto-electrochemical sensors, despite their high specific surface area and high porosity. Here, some of these weaknesses are briefly reviewed, and new developments to address these shortcomings are also provided.

**Poor specificity for analytes:** Conventional MOFs lack the specificity for a single analyte. Non-specificity and subsequent activities from other electroactive analytes or analytes with fluorescence capabilities in the sampling fluid will compromise the accuracy of the results. To increase the specificity of MOF materials for the recognition of a particular analyte, numerous strategies have recently been developed. These strategies include the development of functional MOFs and their combination with bionic enzymatic lock-and-key structures, as well as the introduction of molecular recognition technology such as molecularly imprinted polymers, bioconjugation pairs, aptamers, and antibodies onto MOF structures. In several aspects, MOFs exhibit outstanding superiority over other supporting materials when used as matrices for immobilizing macromolecular biological components such as enzymes or aptamers. This method results in excellent specificity and selectivity of MOF-based opto-electrochemical sensors. Nevertheless, understanding the matrix and immobilization procedures is crucial to maximizing the activity and stability of the immobilized macromolecular entity because the physical and chemical characteristics of these biological entities may change during the immobilization process.

**Low electrical conductivity in conventional MOFs:** Conventional MOFs are well known for having poor electrical conductivity and low carrier mobility, which severely restricts their potential as electrode materials in electrochemical sensors for real-world applications. The main cause of the poor electrical conductivity of MOFs is the widespread use of carboxylate linkers. The electronegativity of the carboxylate oxygen atoms is so high (3.5) that electrons need a higher voltage to pass through the organic linkers. Because of this, the metal d orbitals and oxygen atoms do not overlap well. In order to solve these challenges, research interest in numerous MOF materials with exceptional electrical conductivity and high carrier mobility has increased significantly around the globe. To create such MOFs, a variety of methodologies and strategies have been employed. The formation of MOF-based nanocomposites with carbon-based materials, various metallic/metal oxide nanostructures (e.g., nanoparticles, nano-arrays, nanopillars, and monoliths) are examples of the techniques used to develop conductive MOF-based materials. Additionally, conductive metal nodes (such as Ag^+^, Cu^2+^, and Ni^2+^), redox-active linkers (such as catecholate, 2,3,6,7,10,11-hexaiminotriphenylene, hexaaminobenzene, and ortho-disubstituted benzene) conducting molecules are incorporated into the pores of MOFs [67–73]. MOFs made of different metals, also known as mixed metal MOFs, have recently been produced to dramatically increase the electrical conductivity. Because of the different oxidation potentials and related electron configurations, the bonding between different metal cations in MOFs can improve the electrocatalytic sensing effectiveness of the MOF and boost electrical conductivity.

**Small linear dynamic range of bulk MOFs:** A linear dynamic range, or the range of concentrations where the analytical signals are precisely correlated to the concentration of the analyte in the solution being tested, is one of the crucial factors to evaluate the performance of a robust and reliable sensor. The physicochemical processes on conventional MOFs’ surfaces are weakened because they are typically large and uneven bulk materials, which may limit their performance when employed as opto-electrochemical sensors. As shown in Tables 1–4, some of the bulk MOFs exhibit small linear dynamic ranges. Various research efforts have been made to enhance the functionality of MOFs as opto-electrochemical sensors and expand their linear dynamic range and analyte detection limits. Downsizing bulk MOFs to submicro- or nanoscale sizes is one of these approaches. When compared to bulk MOFs, micro-/nanoscale MOFs (such as nanowires, nanoarrays, nanosheets, nanorods, nanoclusters, and nanofibres) exhibit both the intrinsic properties as well as additional physicochemical characteristics, including less diffusion barriers, shorter diffusion distances for analyte transport, more readily accessible surface active sites, improved conductivity, and higher catalytic efficiency. Also, the number of defects on the outer surfaces of nanoscale hierarchical MOFs is higher. In electrochemical sensing, these characteristics of nanostructured MOFs either enhance their electrocatalytic performance or boost the reaction rate involving analytes bigger than the MOF’s pores.

### General synthesis methods for MOFs

The key factors encouraging the use of MOFs in opto-electrochemical chemo- and biosensing (together with biodegradability, non-toxicity, and facile post-modification) are the positive features already mentioned. A variety of MOFs have been produced using a number of different techniques, and each technique has advantages and disadvantages. The topology, physicochemical characteristics, size, and shape of MOF crystals in micro- and nanoscale regimes are all controlled using various synthetic techniques. To increase product yields, reduce synthesis time, and achieve desirable physicochemical properties, modifications in compositional and process factors are used. The source of metal ions, solution pH, solvent, reactant concentration and molar ratio are compositional parameters that can be changed to control the size and shape of MOF crystals. The crystal size and shape can also be greatly influenced by process variables such as temperature, time, heating source, and pressure. The scope of this review paper does not include detailed synthesis mechanisms, characterisation of MOFs, or a review of each MOF synthesis method. It is strongly advised to read the review articles by Lee et al. [100] and Stock and Biswas [101] for a more comprehensive grasp of MOF synthesis methodologies.

MOFs have mostly been synthesised by conventional electrical heating via small-scale hydrothermal or solvothermal synthesis procedures, which can take hours to several days until complete crystallisation. These techniques are especially well suited for the composition-controlled development of large, high-quality, and extremely crystalline MOFs. Additionally, crystalline phases that are unstable at the melting point can be created using these techniques. In comparison to other traditional synthesis methods, MOFs produced using hydrothermal or solvothermal methods typically have higher chemical purities.

**Figure 13:**
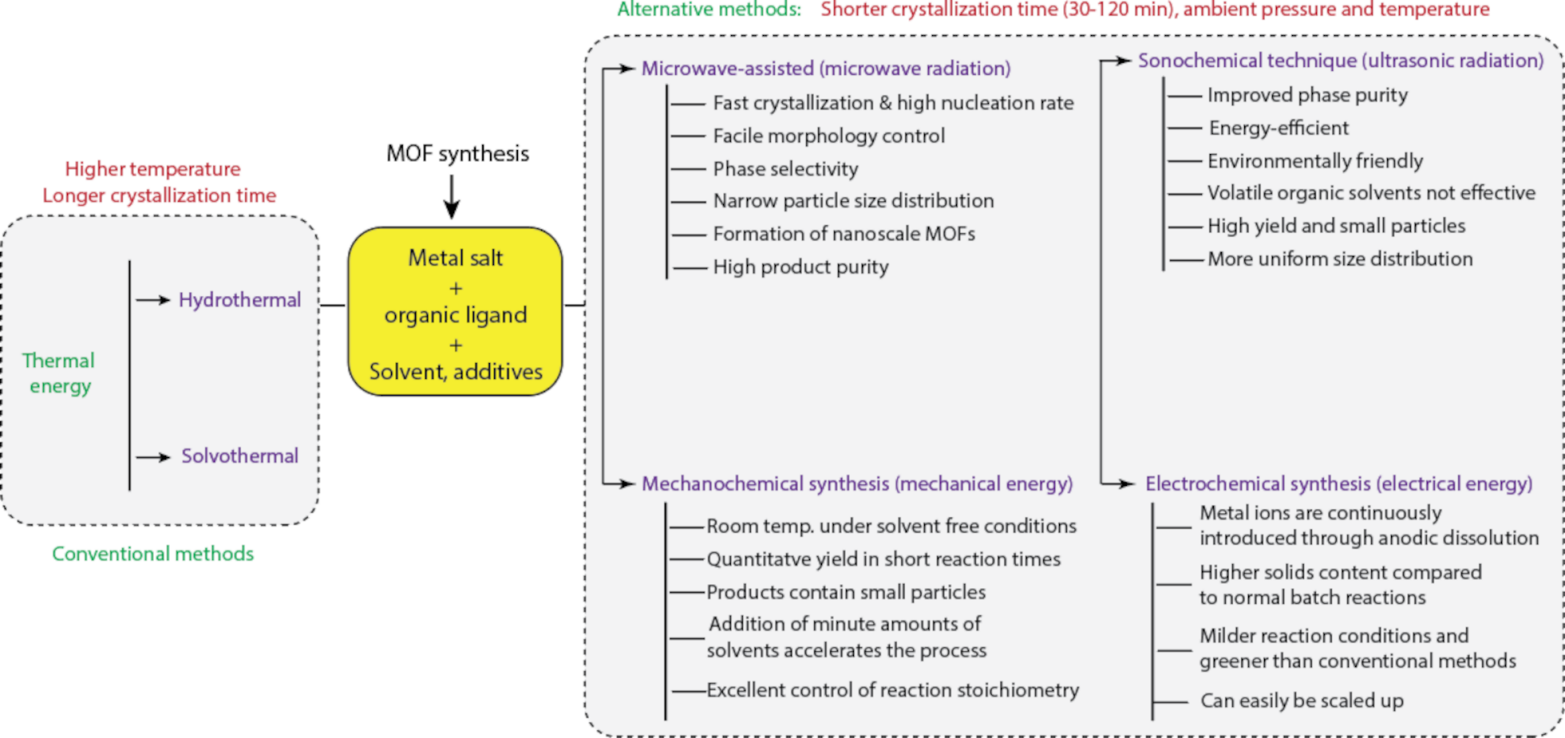
Conventional and alternative synthesis routes for MOFs.

These methods have drawbacks, such as the requirement for expensive autoclaves and the inability to watch the crystal grow if a steel tube is employed, in addition to the lengthy synthesis duration. Various alternative synthesis techniques, including mechanochemical, electrochemical, microwave-assisted, sonochemical, microfluidic, ionothermal, and dry-gel conversion approaches, have since been used to speed up the synthesis time and generate smaller, more homogeneous crystals [67,76,96–97]. Each MOF synthesis method is briefly described in Figure 13, and Lee et al.’s review work [100] includes a detailed demonstration of synthesis methods with faster crystallization periods.

When using the microwave synthesis approach, a high-frequency magnetic field is produced, which can swiftly cause internal heating effects within molecules and cause the temperature of the reaction system to rise steadily and uniformly [77,102] to start chemical reactions. This method can generate MOFs with highly porous structures and lower particle sizes due to rapid crystallisation without causing localised overheating. By using a microwave-assisted technique at 120 °C in a microwave reactor with a microwave power of 100 W, Liu et al. [103] synthesised two-dimensional UiO-67 nanosheets. According to Jhung et al. [98], for instance, the synthesis of MIL-100-Cr using a microwave technique at 220 °C sped up the reaction by 20 times with comparable yield, physicochemical, and textural qualities when compared to the traditional hydrothermal procedure at the same temperature. The combination of the precursors’ quick dissolution and the oxygen–metal networks’ condensation acceleration was thought to be the cause of the rapid synthesis. Abbasi et al. compared a mechanochemical synthesis technique with the sonochemical synthesis of Cu-MOF utilising *N*,*N*-dimethylformamide (DMF) as the solvent [99]. The product yield, specific surface area, and average particle size for the ultrasound-synthesised Cu-MOF are 84.5%, 370.1 m^2^·g^−1^, and about 50 nm, respectively, while they are 76.1%, 1033.8 m^2^·g^−1^, and above 100 nm, respectively, for the microwave-synthesised Cu-MOF.

The synthesis of MOFs using the sonochemical method is quick and continuous, and the rate of synthesis is significantly higher than that of conventional hydrothermal/solvothermal methods. The mechanical effects that the ultrasonic waves have on the medium/solvent as they move through it during the synthesis can help in solvent emulsification, gel liquefaction, and solid dispersion. The MOFs produced by ultrasonic synthesis have homogeneous particle sizes. 2,6-pyridine dicarboxylic acid and zinc nitrate and were used to synthesise Zn-MOF using the ultrasonic-assisted reverse micelle process by Zeraati and co-workers [104]. For the mechanochemical synthesis process, it entails a low-cost mechanical grinding of organic ligands and metal salts, which lessens the effect of solvent volatilization in the environment. The consumption of organic solvents is drastically reduced when thermal energy is replaced with mechanical energy. There are three different ways to make MOFs using the mechanochemical technique, namely neat grinding, which requires no solvent, liquid-assisted grinding, which is rapid but needs a catalytic amount of solvent, and ion- and liquid-assisted grinding, which needs a catalytic solvent and a tiny amount of salt to accelerate MOF crystallisation [67]. Using the mechanochemical synthesis method, Khosroshahi et al. [105] synthesised a heterostructure MOF composite by manually pulverising MOF-808 and NiFe_2_O_4_ in a mortar.

Indirect bipolar electrodeposition, cathode synthesis, anode synthesis, and electrophoresis deposition can all be used to synthesise MOFs electrochemically. Rapid synthesis, benign reaction conditions, and good mesoporous structure are advantages of this approach despite its low yield and propensity for undesirable by-products. MOFs can be made continuously and uniformly, the particle shape may be controlled, and there is a minimal solvent demand [77]. For the electrochemical detection of the insecticide carbendazim in samples of vegetables and fruits, Peng et al. [106] used the electrodeposition approach to quickly and effectively produce a glassy carbon electrode covered with Co-MOFs and carbon nanohorns utilising 1,4-benzenedicarboxylic acid as the organic linker and Co(NO_3_)_2_·6H_2_O as the metal ion source.

Also, during the synthesis of MOFs, the type of solvent used affects the nature of the MOF significantly because of its polarity, protolysis properties, and solubility of the organic ligands. The most often employed solvents include water, ionic liquids, deep eutectic solvents, ethanol, methanol, *N*,*N*-dimethylacetamide, and *N*,*N*-dimethylformamide. Some solvents function throughout the synthesis as ligands, while others serve as both a solvent and a structure-directing agent. To get around issues with varying solubilities of the different starting materials, mixtures of solvents have also been used. According to studies by Loiseau et al. [96] and Senkovska et al. [97] the influence of the solvent was seen for isoreticular MOFs based on the MIL-53 topology, identified as Al-MIL-69 or DUT-4. Using 2,6-naphthalene dicarboxylic acid as a rigid ligand and water as the solvent, Loiseau et al. [96] hydrothermally synthesised Al-MIL-69 at 210 °C for 16 h. The identical metal salt and linker were then employed by Senkovska et al. [97] in a non-aqueous solvent (DMF) at 120 °C for 24 h using a solvothermal method. While the synthesis in DMF as a solvent produced an open-pore MOF that exhibited no flexibility, the synthesis in water produced a nonporous closed structure that could not be opened.

Note that researchers are concentrating on the development of more rapid, continuous, and practical techniques for MOF synthesis in order to meet commercial, specialised application, and industrial needs. Recent research has focused in particular on using optimal synthesis methods to produce a desirable MOF using computational methods.

### MOF-based electrochemical sensors for antibiotics and hormones

Due to their distinctive structural benefits, periodic network architectures, and unsaturated metal coordination sites, MOFs are ideal electrochemical sensing platforms, as was previously mentioned. As a result of these characteristics, MOFs have an enhanced catalytic capacity and can be used to effectively coat electrochemical sensor-based electrodes. Particularly, pristine MOFs with exceptional electroactivities (Cr-MOF, Cu-MOF, Co-MOF, and Ni-MOF) have been directly used to detect various pharmaceutical substances in diverse media. The conductivity and dispersibility of the original MOFs can be increased by the addition of carbon nanomaterials, which enhance the sensors’ electroactivity [76]. Pure MOFs and their composites can be further transformed into MOF-derived carbonaceous and/or metallic nanomaterials and their corresponding composites through the application of a controlled thermochemical process [107], which combines the intrinsic characteristics of MOFs with those of the metals/carbon, making these materials suitable for use as robust and high-performance electrodes for sensors. By integrating functional species into MOFs, it is also possible to develop novel MOF-based electrochemical sensors [108]. These new MOF-based electrochemical sensors will have improved multifunctional properties and selectivity for targeted pharmaceuticals. The active nitro groups found in some regularly used antibiotics, including nitrofurazone, chloramphenicol, and metronidazole, support redox reactions. These antibiotics have the ability to produce redox signals in electrochemical detection when used with MOF-based electrodes.

For example, Rani et al. [50] synthesised a zirconium-based MOF (UiO-66-NH_2_) utilising a solvothermal technique for opto-electrochemical sensing of nitrofurazone. The MOF electrochemical sensing platform demonstrated a good recovery in the range of 97.7–98.4% in lake water samples and a broad linear concentration range for nitrofurazone of 1 × 10^−8^ to 5 × 10^−5^ mol/L. Due to nitrofurazone’s electrochemical activity, its redox activity on MOF-based electrodes was observed as a redox signal. Two reduction peaks and two equivalent oxidation peaks were observed in the cyclic voltammogram of the antibiotic, according to the authors. A one-electron reduction of the nitro group in nitrofurazone to a nitro radical was thought to be the cause of the initial cathodic peak. As a result, a free nitro radical was created, and it was further reduced to a hydroxylamine derivative. It was determined that the nitro radical’s oxidation to nitrofurazone and the oxidation of the hydroxylamine derivative to a nitroso derivative were the causes of two oxidation peaks in the reverse scan. The detection limits for chloramphenicol and metronidazole antibiotics were determined to be 31 and 165 nM, respectively, in a study by Baikeli et al. [109] utilising an iron-doped metal-organic framework (ZIF-8) calcined nanoporous electrode modified with glassy carbon. Nitrogen in the structures of chloramphenicol and metronidazole exerts a redox effect. The Fe dopant in ZIF-8 MOF provides an electron to the nitro group, which is then coupled with the proton donor to generate a reduction product and to produce the electrochemical reaction signal.

A new electrochemical aptasensor based on NH_2_-MIL-101(Fe) MOFs for tetracycline detection was reported by Song and co-workers [70]. The sensor showed a lowest detection limit of 0.01 nM. Investigations showed that the repeatability was good, with the relative standard deviation of ten subsequent measurements being just about 2.3%. Tetracycline recovery rates in actual samples of Donghua University lake and tap water ranged from 89.7 to 102.8%. To produce highly effective electrochemical aptasensors for the detection of ultrasmall traces of penicillin, He et al. [110] synthesised an Ag-based MOF aptasensor. The Ag_2_SiF_6_-MOF-based electrochemical sensor exhibits exceptional penicillin sensitivity and selectivity, with an LOD of 0.849 pg·mL^−1^. The relative standard deviation values are less than 5.0%, and the penicillin recovery rates from the raw milk sample ranged from 99.3 to 104.8%. The findings suggest that the newly developed aptasensor has good accuracy for detecting penicillin in raw milk. Table 1 lists recently published electrochemical MOF-based sensors for detecting antibiotics in various samples, and a review of the findings, significant trends, detection techniques, and technical remarks are provided.

**Table 1:** MOFs for electrochemical sensing of antibiotics in different samples.^a^

Analyte	MOF	Electrode	Method	Real sample	Analytical performance	Ref.

tetracycline (TC)	NH_2_-MIL-101(Fe)	CNF/AuNPs	CV, EIS	tap water lake water	LOD: 0.01 nM linear range: 0.1–10^5^ nM recovery: 92.4–102.8% 89.7–97.4%	[70]
The sensor can be utilized as a new platform for the quantification of TC in an actual water environment. The sensor was made using a combination of electrospinning, pyrolysis, hydrothermal, and electrodeposition processes to increase its sensitivity to the detection of antibiotics.						

oxytetracycline	Ce-MOF@MCA	Au electrode	EIS, CV	milk river water urine	LOD: 17.4 fg·mL^−1^ linear range: 0.1–0.5 ng·mL^−1^ recovery: 101.9–113.6% 94–103.7% 92.6–106.5%	[111]
A simple approach was used to fabricate Ce-MOF@MCA nanohybrids, which were then used as sensitive electrochemical aptasensors to detect oxytetracycline. The MOF-based aptasensors demonstrated outstanding analytical performance with remarkable repeatability, high stability and selectivity, and acceptable performance for detecting antibiotics in different real samples. The performance is attributed to MCA’s large specific surface area, porous nanostructure, highly conjugated degree, and superior electrochemical activity.						

chloramphenicol	IRMOF-8	GCE	SWV, CV	honey	recovery: 96.0–110.0% linear range: 1 × 10^−8^– 1 × 10^−6^ mol·L^−1^ LOD: 2.9 × 10^−9^ mol·L^−1^ sensitivity: 129.1 μA·μmol^−1^·L	[112]
Here, IRMOF-8 was converted to porous carbon using a solvent exfoliation procedure, and the effectiveness was compared against derived porous carbon that had not been exfoliated. Due to its increased surface area and better dispersibility, the exfoliated MOF-based porous carbon displays a noticeably improved electrochemical activity for the detection of chloramphenicol as compared to its parental carbon precursor. The morphology of the electrode material was significantly influenced by the exfoliation solvent type and ultrasonication period. With a signal change below 7%, the sensor’s anti-interference capacity is outstanding in the presence of several metal ions and organic molecules. Additionally, the results demonstrated that the MOF-based sensor has good repeatability, reproducibility, and stability.						

kanamycin and chloramphenicol	UiO-66-NH_2_@M*^n^*^+^/ cDNA M*^n^*^+^: Pb^2+^ and Cd^2+^	GCE	SWV	milk	recovery: 87.4–92.1% for KANA, 86.6–93.0% for CAP linear range: 0.002–100 nM for both KANA and CAP LOD: 0.16 pM for KANA, 0.19 pM for CAP	[113]
Several antibiotics were simultaneously and precisely detected electrochemically using a novel aptamer-metal ions-nanoscale metal organic framework (NMOF). The amine-functionalized MOF had a surface area of roughly 1052 m^2^·g^−1^, and the metal ions loaded into it amplified the electrical signal with great sensitivity and detection limits that were lower than those of ELISA. In terms of selectivity and interference resistance, the MOF-based sensor demonstrated superior performance in food analysis.						

chloramphenicol	MIL-101(Cr)/XC-72	GCE	DPV, CV	honey eye drops milk	recovery: 95–101% 90–101% 95–102% linear range: 1.0 × 10^−8^–2.0 × 10^−5^ M LOD: 1.5 × 10^−9^ M	[114]
A novel MIL-101(Cr)/XC-72 electrode was fabricated to detect chloramphenicol in real samples including milk, honey, and eye drops. The MOF-based sensor showed exceptionally excellent antibiotic recovery from actual samples. The relative standard deviation of the sensor was between 5.2% and 8.0%, and it was also able to maintain 82% of its original current over the course of eight subsequent measurements. Through van der Waals bonds and robust π–π conjugation, the functional groups on the MOF-based hybrid material and the benzene ring structure of MIL-101(Cr) may quickly bind and adsorb the conjugated antibiotic from solutions. The synergistic combination of MIL-101(Cr)’s huge surface area and XC-72’s exceptional conductivity is thought to be the reason for the electrode material’s great performance.						

ciprofloxacin	UiO-66-NH_2_/RGO	GCE	EIS, ASV	tap water lake water	recovery: 96.8–102.4% 105.9–108.6% linear range: 0.02–1 μM LOD: 6.67 nM sensitivity: 10.86 μA·μM^−1^	[115]
A straightforward self-assembly method was used to fabricate NH_2_-UiO-66/RGO composite materials for the Cu^2+^-based anodic stripping voltammetry detection of ciprofloxacin. The complexation reaction between the ciprofloxacin functional groups and Cu^2+^, which yields a stable composite, is the foundation of the sensing strategy. The developed sensor exhibits great selectivity, repeatability, and stability in sensing antibiotics in actual water samples (Dishui Lake in Shanghai and tap water) with satisfactory recovery rates. It is capable of detecting trace quantities of ciprofloxacin. As a result, the disclosed NH_2_-UiO-66/RGO expands the uses of MOF-based materials in electrochemical sensors by overcoming some of their intrinsic disadvantages, such as low electric conductivity and poor water stability.						

vancomycin	P-HKUST-1	GCE	CV	urine, blood serum	linear range: 1–500 nM LOD: 1 nM sensitivity: 496.4 μA·μM^−1^·cm^−2^	[116]
In a single-pot procedure, a Cu-MOF (HKUST-1) was modified using poly(acrylic acid) to produce P-HKUST-1, which was subsequently utilised in an electrochemical sensor for vancomycin detection. P-HKUST-1 is more water-soluble and dispersible than HKUST-1 while maintaining its crystallinity and porosity. Vancomycin can potentially form a complex at the carboxylic acid groups in the linker molecules of the MOF structure.						

tobramycin	Ce/Cu-MOF	CeO_2_/ CuO_x_@mC/AE	EIS, CV	milk human serum	recovery: 97–103% 100.24–103% LOD: 2.0 fg·mL^−1^ linear range: 0.01 pg·mL^−1^ to 10 ng·L^−1^	[117]
In order to develop electrochemical sensors for the sensitive detection of tobramycin in milk and human serum samples, bimetallic Ce/Cu-MOF and its derivatives were synthesised and pyrolysed at various temperatures. The stability of a MOF-based sensor was tested over a two-week period while it was stored at 4 °C, and the results demonstrate that it still exhibits 106% of its initial response. Additionally, after ten reuse cycles, it maintained its initial response with a minimal standard variation of 1.6%, demonstrating the aptasensor’s strong capacity for regeneration.						

tinidazole	Fe-MOF	GCE	EIS, CV, DPV	TDZ tablet human serum urine	recovery: 99% (TDZ tablet) 98.28–108.33% (human serum) 102.92–103.20% (urine) linear range for TDZ: 0.02–525 μM for TDZ LOD: 43 nM LOQ: 143 nM	[118]
To increase the activity and stability of the electrochemical sensor, platinum nanoparticles (Pt NPs) were coated on the Fe-MOF/GCE after the Fe-MOF had been produced chemically. In comparison to the bare electrode, the authors noticed a considerable decrease in the peak separation potential (*E*_p_) and an increase in the current peak at Fe-MOF/Pt-GCE. According to their findings, the electrochemically active surface of GCE was increased by the MOF-based electrode materials by roughly nine times in comparison to the bare electrode. Additionally, even in the presence of interferences, the Fe-MOF/Pt-GCE showed excellent sensing capability for the measurement of TDZ in both biological and pharmaceutical samples.						

ampicillin	Hybrid Co-MOF@TPN-COF	Au electrode	EIS, CV	human serum river water milk	recovery: 95.5–99.9% 98.2–103.4% 96.4–102.6% linear range: 1.0 fg·mL^−1^–2.0 ng·mL^−1^ LOD: 0.217 fg·mL^−1^	[119]
For the detection of widely used β-lactam antibiotics in milk, river water and human serum, a terephthalonitrile-based covalent organic framework and Co-MOF nanoarchitecture were developed. The results show that 15% of the sensor’s initial value was retained over a lengthy duration of 15 days of storage, demonstrating the intended stability of the newly constructed aptasensor. This sensor has demonstrated exceptional performance, and its high surface area, nitrogen-rich groups, and triazine rings have all been associated with this. 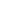						

streptomycin	UiO-66-NH_2_	Au/SPCE	CV, DPV	milk	recovery: 99.4–106% linear range: 0.005–150 ng·mL^−1^ LOD: 2.6 pg·mL^−1^	[120]
After synthesizing Zr-MOF material, a monolayer of thiolated cDNA/aptamer duplexes was employed to immobilize the surface and produce a MOF-based bio-barcode for the detection of streptomycin in milk samples. The sensor’s ability to detect antibiotics through dual-signal amplification is made possible by the immobilized enzyme. In particular, no interference from non-target antibiotics (chloramphenicol, kanamycin, and oxytetracycline) with the detection of streptomycin was found.						

nitrofurazone	AuNPs/UiO-66-NH_2_	SPCE	CV, DPV, EIS	lake water	recovery: 97.70–98.40% linear range: 1 × 10^−8^–5 × 10^−5^ mol/L LOD: 3 × 10^−9^ mol/L	[50]
The solvothermal approach was used to synthesize and characterize a zirconium-MOF. By using an electrochemical-optical dual detection approach, the MOF-based sensor was used to detect the nitrofurazone antibiotic in lake water samples. The MOF was coated with gold nanoparticles to boost the electrochemical signal. High reduction activity towards nitrofurazone was produced by the interaction of porous Zr-MOF and electroactive gold nanoparticles, which boosted the rate at which electrons were transferred between the two materials and the surface area of the electrodes.						

acetaminophen	Ni-doped carbon based on Ni-MOF	GCE	CV, EIS, DPV	human serum urine	recovery: 105.3–107% 97.3–110% linear range: 0.20–53.75 μM LOD: 0.0404 μM	[121]
Following the calcination of Ni-MOF, nickel-doped nanoporous carbon was synthesized. The electrode material exhibits an ordered mesoporous structure, high catalytic active sites and surface area specifically for sensing acetaminophen. The findings showed that, after being maintained at 4 °C for various days, the sensor could be used to accurately measure the presence of acetaminophen in human blood serum and urine samples while maintaining outstanding anti-interference stability and strong reproducibility. After 50 CV cycles, the electrode maintains almost the same current as the first peak current.						

penicillin	Ag(I)-MOF	Ag_2_SiF_6_-MOF/ AuE	EIS, CV	milk	recovery: 99.26–104.80% linear range: 0.001–0.5 ng·mL^−1^ LOD: 0.849 pg·mL^−1^	[110]
Coordination assemblies of a Ag^+^ salt with tri(pyridin-4-yl) amine obtained through a room-temperature diffusion technique were used to fabricate two Ag-based MOFs with different counter anions (SiF62- for 1-based MOF and CH_3_SO_3_ for the 2-based MOF). Compared to Ag-MOF-2 with a greater immobilized quantity of aptamer, the 1-based aptasensor had higher selectivity and specificity for penicillin in the presence of other antibiotic interferences. The structural configuration of the aptamer Ag-MOF-2 was thought to be the cause of its poorer interaction with penicillin.						

enrofloxacin	CoNi-MOF	Au electrode	EIS, CV	milk river water human serum	recovery: 93.5–105.0% 93.3–105.0% 91.2–109.6% LOD: 0.2 fg·mL^−1^ linear range: 0.001–1 pg·mL^−1^	[122]
An aptamer that serves as the recognition component for the rapid identification of antibiotics was used to immobilize a CoNi-based MOF after its synthesis. The CoNi-MOF/Apt electrochemical sensor has additional defects in the MOF skeleton, mixed metal valences, and amino functionality.						

chloramphenicol	CoP-based nanohybrids from ZIF-67	GCE	CV, DPV	milk honey	recovery: 100–102% 98% LOD: 0.044 μM linear range: 0.2–200 μM	[123]
Here, CoPx–N–C hybrids with ordered 3D structures and substantial porosities are synthesized using a cobalt-containing MOF (ZIF-67) as a template. The sensor offers satisfactory repeatability, anti-interference capability, long-term stability, and reproducibility. The CoP2–N–C-based sensor was also useful for chloramphenicol detection in agricultural samples, according to the recovery tests.						

tetracycline	MIL-53 (Fe)	GCE	DPV, CV	tap water river water	recovery: 82.94–112.46% 105.63–115.26% LOD: 0.0260 μmol/L linear range: 0.0643–1.53 μmol/L	[71]
With its ability to detect trace amounts of tetracycline with excellent sensitivity and selectivity, this electrochemical sensor is useful in water bodies. Particularly, when the MIL-based sensor was employed, common organic interferences including phenol, hydroquinone, cations, anions, and catechol, have only a limited influence on the detection of tetracycline.						

ciprofloxacin	HKUST-1	GCE	EIS, CV, DPV	tap water	recovery: 94.6–108% LOD: 0.47 × 10^−9^ mol·L^−1^ linear range: 1 × 10^−8^ to 20 × 10^−6^ mol·L^−1^	[124]
The Cu-MOF was synthesized using a hydrothermal process, and its electrochemical detection of ciprofloxacin was shown to be sensitive and selective. The good recoveries attained further show the possible use of the developed electrode for the investigation of real-life water samples. Also take note of the fact that the peak current showed no discernible drop after 28 days of dry storage, demonstrating significant stability of the developed sensor.						

monensin	Zn/Ni-ZIF-8	GCE	CV, DPV	milk	recovery: 94.4 to 112.0% LOD: 0.11 ng·mL^−1^ linear range: 0.25–100 ng·mL^−1^	[125]
To develop a reliable electrochemical sensor electrode material, the Zn/Ni-bimetallic-based ZIF-8 MOF was synthesized, pyrolyzed at 800 °C, and modified with graphene and AuNPs. The high porosity of the MOF and the superior electrical conductivity of graphene and AuNPs, among other synergistic effects, are advantageous to the sensor. Monensin in milk was detected using the sensor. Additionally, due to the precise conjugation between the antibodies and monensin, AuNPs functioned as a support to immobilize the anti-monensin monoclonal antibodies and exhibit great selectivity with good anti-interference capabilities.						

sulfamethoxazole	ZIF-67	CPE	CV, DPV	tap water river water urine	recovery: 98.0–101.3% 98.75–103.3% 97.1–102.0% LOD: 5.0 nM linear range: 0.01–520.0 µM	[126]
The basis of this electrochemical sensor is the employment of a carbon paste electrode modified with an ionic liquid and nanocomposite made of Fe_3_O_4_ and ZIF-67 MOF. This electrode, which has strong selectivity and sensitivity characteristics, could be utilized extensively for the determination of sulfamethoxazole in actual samples. The voltammetric sensor’s performance was enhanced by the ionic liquid to enable the precise and highly sensitive detection of electroactive substances in pharmaceutical and biological samples.						

chloramphenicol	ZIF-8	GCE	CV, DPV	milk honey	recovery: 101.4–106% 98.9–102.4% LOD: 0.25 μM linear range: 1 to 180 μM	[127]
To develop a hybrid electrochemical sensor for chloramphenicol detection, porous carbon was produced from ZIF-8 by carbonization at 800 °C, modified with graphene oxide, and then doped with nitrogen. The synergistic effects of C–N active sites of ZIF-8 and high electrical conductivity of rGO are responsible for the MOF-based sensor’s effective electrocatalytic activity.						

chloramphenicol and metronidazole	Fe/ZIF-8	GCE	EIS, CV, LSV	urine milk CAP eye drops MNZ tablets	recovery: 106.4–109.3% for CAP 107.8–109.5% for MNZ 31.2–36.7% for CAP 93.2–105.4% for MNZ 91.4–94.6% for CAP 98.2–101.9% for MNZ 96.3–105.2% for CAP 98.3–105.5% for MNZ	[109]
					linear range: 0.1–100 μM for CAP 0.5–30 μM for MNZ LOD: 31 nM for CAP 165 nM For MNZ	
To produce nanoporous nitrogen-doped carbon nanoparticles, an iron-doped MOF (Fe/ZIF-8) was first synthesized at ambient temperature and subsequently carbonized under a nitrogen environment. These nanoparticles are employed as electrode materials for electrochemical detection of antibiotics. Due to the abundant nitrogen content, distinctive porous structure of the nanoporous components, catalytic activity of Fe atoms, and large surface area, the MOF-based electrochemical sensor displayed exceptional electrochemical performance.						

metronidazole	ZIF-67	GCE	CV, EIS, DPV	MNZ tablets tap water	recovery: 96.5–104.2% 94.0–98.0% linear range: 0.5 to 1000 μM LOD: 0.05 μM	[128]
Graphite oxide was utilized in this case as a substrate for in-situ assembly with the zeolitic imidazole framework ZIF-67. The composite was then transformed via pyrolysis into Cobalt/N-co-doped carbon MOF-based polyhedrons for the detection of the antibiotic metronidazole. The sensor, which had good electrical conductivity, abundant active sites, and hierarchically open pores, performed exceptionally well with optimized amount of GO were utilized. It also had great selectivity, good repeatability, and satisfactory recoveries.						

^a^MCA: melamine and cyanuric acid; rGO: reduced graphene oxide; LSV: linear sweep voltammetry; CV: cyclic voltammetry; DPV: differential pulse voltammetry; EIS: electrochemical impedance spectroscopy; GCE: glassy carbon electrode; SPCE: screen-printed carbon electrode; CPE: carbon paste electrode.

Numerous MOF-based electrochemical sensors have also been used to detect hormones at various concentrations. Some of these hormones have electrochemical properties that make it easier for MOFs to detect them, while analyte recognition molecules are typically added to MOF materials to enhance the sensing and selectivity of the sensor materials. For instance, Li et al. [129] reported the development of a copper-based MOF electrochemical sensor for the detection of dopamine, a significant neurotransmitter. The as-synthesized MOF electrode sensor showed strong selectivity for dopamine in real samples when operated under optimal conditions, with a detection limit as low as 1.5 × 10^−7^ M and a linear response range of 5.0 × 10^−7^ to 1.0 × 10^−4^ M. By immobilizing a monoclonal antibody specific for thyroxine onto a Cu-MOF modified with polyaniline, Mradula et al. [130] reported a label-free electrochemical immunosensor for the detection of the hormone thyroxine. The MOF-composite immunosensor demonstrated high selectivity, a rapid detection time of 20 min, a detection limit of 0.33–0.17 pM, and a linear range of 10–10^5^ pM. In order to detect the follicle stimulating hormone (FSH) antigen, Palanisamy et al. [131] created amine-functionalized Fe-containing MOFs and then bound them with FSH antibodies. According to the study, the estimated LOD for the MOF-based immunosensor material for FSH was 11.5 and 11.6 fg/mL for serum and buffered solutions, respectively. MOF-based electrochemical immunosensors are fast, precise, portable, and typically disposable, requiring very little sample volume for automated hormone detection as compared to sophisticated, expensive, and time-consuming methodologies and advanced equipment. Recent studies on MOF-based electrochemical sensors for the detection of various types of hormones are reviewed, summarized, and provided in Table 2. These studies include important parametric conditions, detection mechanisms, and significant results.

**Table 2:** MOFs for electrochemical sensing of hormones in different samples.^a^

Analyte	MOF	Electrode	Method	Real sample	Analytical performance	Ref.

dopamine	HKUST-1	GCE	DPV, CV, EIS	injection samples	recovery: 99.40–103.0% linear range: 5.0 × 10^−7^−1.0 × 10^−4^ M LOD: 1.5 × 10^−7^ M	[129]
HKUST-1’s large specific surface area and excellent adsorption capability, which can adsorb more dopamine on the electrode surface, are responsible for the MOF-based sensor’s excellent current response. The electron transport between dopamine and the electrode surface can be facilitated by the MOF’s excellent electrical conductivity and three-dimensional pore structure. Additionally, the catalytic oxidation of dopamine was facilitated by the active metal nodes and ligands equipping HKUST-1 with redox activity.						

thyroxine	Cu-MOF	SPCE	DPV, CV	fetal bovine serum	recovery: 95–102% linear range: 10–10^5^ pM LOD: 0.17 pM (0.13 pg/mL) for DPV 0.33 pM (0.25 pg/mL) for CV	[130]
The immunosensor was developed by screen-printing a modified Cu-MOF and polyaniline composite carbon electrode with a monoclonal antibody that is specific to thyroxine. The Cu-MOF@PANI@Ab immunosensor was discovered to be extremely repeatable and unaffected by the presence of interfering chemicals. The particular interaction between the antibody and antigen on the immunosensing platform served as the basis for the detection method.						

follicle-stimulating hormone	H_2_N−Fe-MIL-101	nickel foam	EIS, CV	—	recovery: — linear range: 100 ng/mL–100 pg/mL LOD: 11.6 fg/mL for buffered solutions 11.5 fg/mL for serum solutions	[131]
The electrostatic interactions between the MOFs’ amino groups and the hormone’s negatively charged carboxylic acid or hydrogen bonding are the basis for the detection process.						

dopamine	Cu-MOF	GCE	CV, DPV	human serum urine	recovery: 98% 98% linear range: 1–50 μM LOD: 0.21 μM	[132]
Graphene oxide was used as the supporting carrier in the preparation of the Cu-MOF-based composite. The composite has good water dispersibility and stability due to the strong hydrophilicity of GO and its interaction with the Cu-MOF. Regarding the detection of dopamine in the presence of common inorganic ions, the Cu-MOF/GO displayed great sensitivity and low interference.						

indole-3-acetic acid	Zn-MOF	GCE	SWV, CV, EIS	peanuts corn	recovery: 98.90–102.22% 98.95–103.92% 99.48–106.02% linear range: 2.5 pg·mL^−1^–5 ng·mL^−1^ LOD: 1.4 pg·mL^−1^	[133]
In this case, a toluidine blue-functionalized MOF (TB@MOFs) thin film was created using an in situ, one-step reduction technique. A MOF-based immunosensor for the detection of plant hormones was developed by covalently immobilizing antibodies onto the TB@MOF using chitosan. With relative errors of less than 4%, the sensor showed good recovery.						

estradiol	Cu-MOF	CPE	CV, DPV	water samples	recovery: 96.5–101% linear range: 5.0–650.0 nM LOD: 3.80 nM	[134]

Only the irreversible oxidation wave was seen for the Cu-MOF during the detection of estradiol. Increases in pH caused a negative shift in the oxidation peak potential, indicating that protons are involved in the oxidation process. The results showed that the estrogen’s oxidation signals were pH-dependent.						

estradiol	Zr porphyrin MOF	GCE	CV, DPV	urine serum samples	recovery: 98%–103% linear range: 0.01–210 μM LOD: 0.5 nM	[135]
Using a hydrothermal method, Zr-porphyrin MOF with a PCN-222 structure was synthesized. In order to fabricate the AuHPCN-222/GCE electrochemical sensor for estradiol detection, the MOF was functionalized with an optimal amount of subnanometer-sized Au(0) and then coated on glassy carbon. The electrocatalytic sites and charge transfer are greatly improved by the functionalized zero-valent Au within the MOF via a hopping mechanism.						

estradiol	MIL-53	GCE	CV, DPV	water sample	recovery: 96.9–103.9% linear range: 10^−14^–10^−9^ mol·L^−1^ LOD: 6.19 × 10^−15^ mol·L^−1^	[136]
Prussian blue (PB), carbon nanotubes (CNT), and MIL-53 (MOF) were used to create a hybrid electrochemical sensor that can detect 17-estradiol. The hybrid was combined with pyrrole-based molecularly imprinted polymer (MIP) to construct MIP-PB-MIL-CNTs, which further increased selectivity. The great performance of the sensor was due to the huge specific surface area of the MOF, outstanding electrocatalytic activities, and conductivity of the CNTs and PB nanoparticles.						

estrone	AuZn-MOFZn-MOF: (ZIF-8)	CPE	CV, DPV	water sample	recovery: 93.0–103.5% linear range: 0.05–5 μM LOD: 12.3 nM	[137]
At room temperature for 12 h, bimetallic MOF (AuZn-MOF) was synthesized. the MOF host was then coated with Au nanoparticles, dramatically increasing its ability to transmit electrons and the amount of electrochemically active surface area. The electrochemical activity toward the oxidation and sensing of the hormone-disrupting chemical Estrone was markedly enhanced in the hybrid Au NPs@AuZn-MOFs as compared to the bimetallic AuZn-MOF.						

epinine	ZIF-67	SPE	DPV, CV, LSV	urine	recovery: 97.5–103.3% 97.3–102% linear range: 9.0 × 10^−8^ M–5.0 × 10^−4^ M LOD: 2.0 nM	[138]
To create a GO/ZIF-67 electrochemical sensor for the detection of epinine, ZIF-67 MOF was modified with graphene oxide. The electrochemical reactions had the same number of protons and electrons, as indicated by the pH influence on epinine detection, and the scan rate effect revealed the diffusion-controlled method.						

serotonin	ZIF-67	GCE	CV, EIS, amperometry	human blood serum urine	recovery: 98.35–99.7 98.90–99.45 linear range: 0.049 to 800 μM LOD: 7 nM	[139]
To develop ZIF-67/MWCNT as a sensor material, a zeolite imidazole framework-based MOF was combined with multiwall carbon nanotubes. The oxidation of serotonin to a quinone imine, which involves two electrons and protons, is the basis for the detection mechanism. The electrochemical investigations clearly show that ZIF-67/MWCNT with increased surface area and enriched electrocatalytic activity resulted in higher sensing performance.						

dopamine	ZIF-67 (nanopinnas)	GCE	CV, DPV	DA injection samples	recovery: 96.3–103.4% linear range: 0.1–100 μM LOD: 0.05 μM	[29]
A composite material (Ag-ZIF-67p) was synthesized using an ultrasonication approach by fabricating a ZIF-67-based MOF and depositing Ag nanoparticles on it. Due to ZIF-67’s large surface area and porosity, as well as Ag nanoparticles’ high conductivity and strong catalytic activity, the composite MOF-based material displayed improved electrocatalytic activity toward dopamine and acetaminophen.						

dopamine, serotonin	ZIF-8	GCE	CV, EIS, DPV	human serum albumin	linear range 0.1–50 μM for DA 0.1–25 μM for ST LOD: 0.03 μM for DA 0.007 μM for ST	[140]
A ZIF-8 assembly was carefully enclosed with gold nanorods to produce gold nanorod-doped ZIF-8 (Au@ZIF-8) nanostructures. It is believed that the high conductivity of Au nanorods and the extensive catalytic sites of ZIF-8 are combined to improve the electrochemical sensor’s performance.						

adrenaline	CoMn-ZIF	GCE	CV, EIS, DPV	human serum	recovery: 97.51–102.53% linear range: 5–1000 μM LOD: 0.22 μM	[141]
This study developed a carbon nanofibre-modified zeolitic imidazolate framework electrochemical sensor for the ultra-sensitive detection of adrenaline. The high electrocatalysis of CoMn-ZIF and the strong conductivity of carbon nanofibres in the MOF-based composite material can increase the reaction activity and the sensing effectiveness of adrenaline.						

dopamine	ZIF-67	GCE	CV, EIS, amperometry	—	linear range: 0.25–1216.25 μM LOD: 0.052 μM	[142]
ZIF-67/rGO, a composite material made of a cobalt-based zeolitic imidazolate framework, serves as an electrochemical sensor for dopamine. The interaction between the sensor and the target analyte can be aided and facilitated by the MOF’s large surface area, the rGO’s electrostatic attraction to the positively charged dopamine, and the interaction between the aromatic groups of the rGO and the aromatic groups of dopamine through π–π bonds.						

dopamine	UIO-66-NH_2_	GCE	EIS, DPV, CV	human serum	recovery: 101.2–103.5% linear range: 1–250 fM LOD: 0.81 fM	[143]
UIO-66-NH_2_ was doped with the optimal amount of Ti_3_C_2_ through hydrogen bonding to produce a MOF-based Mxene membrane. The electrode material was synthesized with a hierarchical cave-like architecture, a significant specific area, great film-forming capabilities, and an exceptional electronic conductive network, which expands its use in electrochemical sensors.						

dopamine	Ni-MOF	GCE	DPV	dopamine hydrochloride injection	recovery: 95.9–104.3% linear range: 0.2–100 μmol L^−1^ LOD: 60 nmol L^−1^	[144]
The Ni-MOF was synthesized using the ionothermal synthesis approach, and the addition of ionic liquid as a template increased the electrochemical sensing performance toward dopamine. The Ni-MOF’s huge accessible surface area, numerous activity sites, and the ionic liquid’s excellent electrical conductivity functioned together to provide the MOF with exceptional electrocatalytic performance.						

adrenaline, serotonin, tryptophan	ZIF-67@ZIF-8	GCE	CV, DPV, EIS	rat brain tissue rat serum	recovery: ST: 97–101.3% adr: not detected recovery: ST: 93.5–97.5% adr: 94.5–101.8% linear range: 0.3–6 μM for Adr 0.1–10 μM for ST LOD: 0.09 μM for Adr 0.03 μM for ST	[145]
A self-cleaning electrochemical electrode sensor based on the zeolite imidazole framework (ZIF-67@ZIF-8) was produced by vaporizing the precursor to develop a hydrophobic layer of polydimethylsiloxane on the carbonized MOF. Self-cleaning electrodes and ratiometric electrochemical detection of different hormones were coupled in the newly developed MOF-based innovative sensing platform.						


adrenaline	ZIF-67	three-dimensional graphene fibre	CV, DPV	human serum	recovery: 99.7–116.3% linear range: 0.06–95 μM LOD: 0.02 μM	[146]
A simple one-pot electrodeposition self-assembly technique was employed to embed a nitrogen-rich, carbon-coated MOF (ZIF-67) in a three-dimensional graphene fibre, which served as a high-performance electrode for the non-enzymatic detection of adrenaline.						

catechol	MIL-101(Cr)	CPE	DPV, EIS, CV	lake water	recovery: 98.1–103.2% linear range: 10 to 1400 μM LOD: 4.17 μM	[147]
For the modification of a carbon paste electrode, reduced graphite oxide (rGO) was added to a metal-organic framework (MIL-101(Cr)). The resulting electrodes showed remarkable sensitivity and reproducibility in the simultaneous electrochemical quantification of catechol and identification of hydroquinone by taking advantage of the electrical conductivity of rGO and the huge surface area of MOF.						

catechol	Zn-MOF	CPE	DPV, EIS, CV	river water	recovery: 88–102% linear range: 0.050–100 μM LOD: 1 nM	[148]
A metal-organic framework (Zn-MOF), gold nanoparticles and nitrogen-doped graphite were combined to modify a carbon paste electrode and use it as an electrochemical sensor for dihydroxybenzene isomers (hydroquinone, catechol, and resorcinol).						

catechol	Ce-MOF	GCE	CV, DPV	river water	recovery: 101.5–103% linear range: 5–50 μM LOD: 3.5 μM	[149]
Nanocomposites made of Ce-MOF and carbon nanotubes were produced and then post-treated with a solution containing a mixture of hydrogen peroxide and sodium hydroxide. The post-treatment induced the core atom Ce to undergo partial oxidation, converting it from trivalence to tetravalence. The simultaneous detection of hydroquinone and catechol was achieved using the nanocomposite-based electrochemical sensor, which demonstrated excellent performance attributed to the synergistic interaction between the high surface area of MOF, the high valence of Ce, and the high electrical conductivity of carbon nanotubes.						

catechol	ZIF-8	GCE	DPV, EIS, CV	tap water	recovery: 95–102% linear range: 0.5–70 μM LOD: 0.076 μM	[150]
The ultrasonication technique was used to produce a ZIF-8 (MOF). To develop ZIF-8C@rGO nanocomposite electrode material for the electrochemical measurement of hydroquinone and catechol, reduced graphite oxide (rGO) and carbon from MOF were mixed. Due to the interaction between the self-templated ZIF-8C anchors and the graphitized rGO substrate, the MOF-based electrode material exhibits noticeably better selectivity and sensitivity than its ZIF-8C counterpart.						

catechol	C-ZIF-67	GCE	CV, EIS, DPV	water sample	recovery: 99.6–100.12% linear range: 1–200 μM LOD: 1.0 μM	[151]
To fabricate C-ZIF-67/PAN for the electrochemical detection of hydroquinone and catechol, the synthesized ZIF-67 (MOF) was pyrolyzed at 800 °C in a N_2_ environment after being decorated with polyacrylonitrile using an electrospinning technique. To increase the porous carbon’s electrochemical activity for sensing, the polymer that was electrospun onto the MOF added enough nitrogen to the sample and increased the specific surface area.						

catechol	Cu-MOF	GCE	CV, EIS, DPV	river water	recovery: 97.53–101.67% linear range: 12.5–900 μmol∙L^−1^ LOD: 1.65 μmol∙L^−1^	[152]

Using a hydrothermal process, Cu-MOF and graphene oxide aerogel composite was produced, and used as electrochemical sensors to detect trace amounts of catechol. Combining Cu-MOF, which has great catalytic activity, with graphene oxide aerogel, which has excellent electrical conductivity and stability, resulted in a complex with an enclosed structure that boosted the benefits of both materials while making up for their defects. Comparing the prepared modified electrodes to the bare GCE, they showed better detection performance.						

^a^LSV: linear sweep voltammetry; CV: cyclic voltammetry; DPV: differential pulse voltammetry; EIS: electrochemical impedance spectroscopy.

### MOF-based fluorescent sensors for antibiotics and hormones

Some key intrinsic properties of MOFs, particularly those with lanthanide metal nodes, include higher quantum yields, a larger Stokes shift, and a longer lifetime. These characteristics make MOFs attractive fluorescence sensors for detecting various analytes. Recently, increasing research efforts have been focused on the fabrication of novel fluorescent MOFs for detecting trace amounts of hormones and antibiotics. Organic ligands commonly serve as recognition sites for the precise detection of analytes and give rise to the fluorescence of MOFs [153–158]. By lowering non-radiative relaxation or improving the ligand-to-metal charge transfer activity, organic ligand modifications that functionalize MOFs yield high fluorescence emissions [155].

Additionally, the immobilisation of hydrophilic groups (such as RCOO^−^, –NH_2_ and RSO_3_^−^) onto the organic ligands might improve the water solubility and structural stability of MOFs, expanding the range of potential uses for them in aqueous environments. For instance, by synthesising a Eu-doped NH_2_-MIL-53(Al) nanostructured material, Chen et al. [156] developed an antibiotic detection probe with fluorescence. Tetracycline was added to the sensor solution, and the antenna effect produced by coordinating tetracycline with Eu^3+^ enhanced the fluorescence intensity at 616 nm, the characteristic emission peak of Eu^3+^, whereas the fluorescence of the MOF at 433 nm was quenched by Förster resonance energy transfer between the tetracycline–Eu^3+^ complex and the MOF. As a result, tetracycline was detected effectively thanks to this shift in the fluorescence signal ratio. Table 3 and Table 4 highlight recent studies using MOF as fluorescence sensors for the detection of hormones and antibiotics. These tables review, enumerate, and provide information on the detection procedures, types of MOFs, and real samples in which the analytes have been identified.

**Table 3:** MOFs-based fluorescence sensor for antibiotics in different samples.^a^

Analyte	Sensing materials	Ligand/reagent	Real sample	Analytical performance	Mechanism	Ref.

tetracycline,. (TC), doxycycline (DOX), oxytetracycline (OTC), chlortetracycline (CTC)	Al-MOF@Mo/Zn-MOF	NH_2_-BDC	water sample milk	recovery: 100.21–106.40% for TC 87.07–106.69% for DOX 88.86–99.34% for OTC 89.83–100.48% for CTC 107.01–111.67% for TC 103.21–110.95% for DOX 97.40–103.53% for OTC 103.2.5–116.44% for CTC linear range: 0.001−53.33 μM for TC 0.001−46.67 μM for DOX 0.001−53.33 μM for OTC 0.001−53.33 μM for CTC LOD: 0.53 nM for TC 0.56 nM for DOX 0.58 nM for OTC 0.86 nM for CTC *K*_sv_: 3.51 × 10^4^ M^−1^ for TC 3.32 × 10^4^ M^−1^ for DOX 3.22 × 10^4^ M^−1^ for OTC 2.19 × 10^4^ M^−1^ for CTC	PET, IFE, H-bonding interaction between TC and MOF	[153]
To achieve remarkable high-performance tetracycline detection, a three-dimensional hierarchical dual-MOF heterostructured nanomaterial was fabricated. The MOF fluorescence was quenched as a result of the organic ligands of the dual-MOF being modified with NH_2_ functional groups for specific recognition toward the analyte.						

metronidazole	Eu(III)-MOF	1,10-phenanthro- line	—	linear range: 0.06–0.17 mM LOD: 2.75 μM *K*_SV_: 2.39 × 10^4^ M^−1^	fluorescence quenching	[154]
MOFs based on lanthanides (La, Eu, Gd, and Tb) were synthesized using a hydrothermal method. The Eu-MOF revealed a polyhedral three-dimensional network structure, bright red emission, and a high 75.6% fluorescence quantum yield. Notably, the Eu-MOF has great stability in water across a wide pH range (pH 3–13) and is thermally stable in air, extending its application for antibiotic detection. Along with other nitro antibiotics, the Eu-MOF also demonstrated the sensitive and selective detection of Fe^3+^/Cr^6+^ in an aqueous solution.						

tetracycline	CBO@ZIF-8	2-methyl- imidazole	—	linear range: 0−45 μΜ LOD: 26 nM	fluorescence enhancement	[155]
Hydrothermal synthesis of zeolitic imidazolate framework-8 (ZIF-8) was followed by CuBi_2_O_4_ (CBO) modification to produce MOF-based fluorescence sensors with good stability, a high surface area, and inherent catalytic capability. At an emission wavelength of 517 nm, pure CBO@ZIF-8 and TC displayed essentially no fluorescence, but as soon as TC was added to the sensor solution, fluorescence was amplified considerably.						

tetracycline	Eu^3+^/NH_2_-MIL-53(Al)	NH_2_-H_2_BDC	tap water	recovery: 95.03–103.26% linear range: 0.5–60 μM LOD: 0.16 μM	FRET	[156]
Hydrothermally produced NH2-MIL-53(Al) MOF-based nanosheets were subsequently doped with Eu^3+^. The antibiotic-induced FRET effect was seen in the Eu modified MOF. As an energy donor, the MOF’s fluorescence intensity was reduced, however as an energy acceptor, the fluorescence intensity of the TC-Eu-doped MOF complex is increased. Thus, tetracycline detection was made possible by altering the ratio of fluorescence signals.						

chloramphenicol	PCN-222	tetrakis(4-carboxy- phenyl)porphyrin (H_2_TCPP)	milk shrimps	recovery: 93.37–105.60% 91.25–104.47% linear range: 0.1 pg·mL^−1^– 10 ng·mL^−1^ LOD: 0.08 pg·mL^−1^	FRET, PET	[157]
For the very effective detection of chloramphenicol, a zirconium-porphyrin MOF (PCN-222) fluorescence quencher and an aptamer tagged with a fluorescent dye were combined to produce a ratiometric fluorescent sensing material. The π-conjugated ligands, strong electron-accepting Zr ions, and PCN-222 with rich mesoporous structure and high surface area were the sources of the quenching mechanism. Together, they had a significant impact on both the rate and sensitivity of the analysis.						

metronidazole	Cd-MOF	phenanthroline	lake water tap water urine	recovery: 99.3–101.3% 98.5–99.2% 98.8–02.2% LOD: 0.10 μM *K*_SV_: 1.56 × 10^5^ M^−1^	IFE	[158]
As a fluorescent antibiotic sensor, polyvinylpyrrolidone-assisted synthesis was used to produce two-dimensional, highly water-stable cadmium-based MOF nanosheets. When taking into account the HOMO and LUMO locations of the antibiotic and the sensing material, the quenching mechanism is attributed to the inner filter effect.						

metronidazole	Zn-MOF	4,4′-oxybis(benzoic acid) (oba) and 4,4′-bipyridine (4,4′-bpy)	river water rain water tap water urine	recovery: 98.28–105.9% 98.27–101.1% 99.03–100.9% 100.7–101.4% LOD: 0.81 mg·L^−1^ *K*_SV_: 6.44 × 10^3^ L·mol^−1^	IFE	[159]
Through a facile solvothermal process, a new fluorescent Zn-MOF was successfully developed at 130 °C over 48 h. The MOF emitted a violet light based on a ligand-to-metal charge transfer (LMCT) mechanism, which was very specifically quenched in aqueous systems by aromatic nitrophenols or metronidazole.						

dimetridazole (DTZ), nitrofurantoin (NFT)	Tb-MOF	urea-functionalized tetracarboxylate ligand	tap water river water	recovery: 101.0–101.2% for NFT 97.6–104.9% for DTZ 107.1–108.4% for NFT 97.7–104.4% for DTZ linear range: 0–82.6 μM for NFT 0–200 μM for DTZ LOD: 0.41 μM for NFT 1.39 μM for DTZ *K*_SV_: 1.9 × 10^4^ M^−1^ for NFT *K*_SV_: 1.0 × 10^4^ M^−1^ for DTZ	PET	[160]
A urea-functionalized tetracarboxylate ligand was used in a simple solvothermal technique to easily fabricate a three-dimensional porous Tb(III)-based MOF. As a result of the antenna ligand’s partial energy transfer to Tb^3+^, the Tb-MOF displayed dual luminescence (also known as a “turn-off” or “turn-on” effect).						

nitrofurantoin	ZTMOF-1	terpyridine	tap water lake water	recovery: 92.4–106% 92.4–108% LOD: 0.175 μM *K*_SV_: 7.055 × 10^4^ M^−1^	energy transfer and charge transfer	[161]
The terpyridine-based zinc-MOF (ZTMOF-1) was synthesized and decorated with a phenyl group that interacted with the guest species. The as-synthesized ZTMOF-1 can function as a dual-responsive sensor that not only exhibits good performance for pyrophosphate in differentiation from other phosphorus oxides in both aqueous solution and physiological environment, but also can effectively sense nitrofurantoin in various samples with good reusability and high sensitivity and selectivity.						

nitrofurazone (NFZ), nitrofurantoin (NFT); furazolidone (FZD)	Eu-MOF	H_3_BTCTB: benzenetriyl- tris(carbonyl- imino)]tris- benzoate	–	linear range: 0–59 μM for NFZ 0–63 μM for NFT 0–77 μM for FZD LOD: 0.67 ± 0.08 μM for NFZ 0.60 ± 0.06 μM for NFT 0.51 ± 0.06 μM for FZD *K*_SV_: 1.27 ± 0.03 × 10^4^ M^−1^ for NFZ *K*_SV_: 2.10 ± 0.03 × 10^4^ M^−1^ for NFT *K*_SV_: 1.72 ± 0.04 × 10^4^ M^−1^ for FZD	IFE, PET	[162]
Tris-benzoic benzoate (H3BTCTB) was used as the antenna ligand to self-assemble with Eu^3+^ salt for a long-lasting and reliable fluorescence sensor in this study. Three amide bonds connected to the antenna ligand acted as Lewis base and hydrogen-bonding sites and improved the sensing performance in water systems in addition to rigid benzoate moieties which bind the Eu^3+^ ions and enhanced the stability.						

nitrofurazone (NFZ), nitrofurantoin (NFT)	Zn-MOF	5-(4-carboxy- phenoxymethyl)- isophthalic acid (H_3_Cbbi) and 1,2-bis(4-pyridyl) ethylene (bpee)	—	recovery: linear range: LOD: 0.22 ppm for NFZ 0.15 ppm for NFT 48.8 μM for NFT 67.6 μM for NFZ *K*_SV_: 54348 M^−1^ for NFT *K*_SV_: 37448 M^−1^ for NFZ	PET, IFE	[163]
For the ultra-sensitive detection of nitrofuran antibiotics, Zn(II)-based MOFs were produced hydrothermally and used as chemosensors. The various nitrogen donor ancillary ligands in the structure demonstrated distinct topological structures and fluorescence characteristics as a result of the different spacers they contained.						

nitrofurazone (NFZ), nitrofurantoin (NFT), furazolidone (FZD)	Al-MOF	NH_2_-BDC	milk	recovery: 88.14–126.21% linear range: 0.5–80 μM for NFT 1–70 μM for NFZ 0.5–80 μM for FZD LOD: 0.53 μM for NFZ 0.838 μM for NFT 0.583 μM for FZD	IFE	[164]
Al-MOF nanosheets containing luminescent fusiform Al(III) were successfully made using a one-step hydrothermal process. The sensor’s benefits were rapid detection, increased sensitivity, superior stability, and strong anti-interference capabilities. This research opens up a new avenue for the visible and sensitive detection of nitrofurans, with potential uses in food sample analysis.						

ciprofloxacin (CIP), ofloxacin (OFLX)	Eu-MOF	5-(1*H*-pyrazol-3- yl)isophthalic acid (H_2_PIA)	–	linear range: LOD: 0.693 ppb for CIP 0.802 ppb for OFLX *K*_SV_: 22407 M^−1^ for CIP *K*_SV_: 22751 M^−1^ for OFLX	PET, dynamic quenching process	[69]
Using a hydrothermal approach, a luminescent Eu-MOF with a distinctive two-dimensional interconnected architecture was synthesized. It displayed excellent fluorescence properties as well as high chemical stability in an aqueous solution for fifteen days.						

nitrofurazone (NFZ), nitrofurantoin (NFT)	Tb-MOF@PMMA	aminoisophthalic acid and CH_3_COONa	bovine serum	recovery: 94.8–104.4% for NFT linear range: 0–60 μM LOD: 0.30 μM for NFT 0.35 μM for NFZ *K*_SV_: 2.8 × 10^4^ for NFZ *K*_SV_: 4.0 × 10^4^ for NFT	IFE	[165]
The processability of poly(methyl methacrylate) polymer and Tb-MOF were coupled when lanthanide-MOF was synthesized and filled with mixed matrix membranes (MMMs). The Tb-MOF-MMMs demonstrated very high stability in water with a wide pH range and characteristic blue emission for highly selective and sensitive detection of nitrofuran antibiotics while remaining unaffected by metal ions and anions that coexisted in the solution as well as by other common antibiotics.						

chloramphenicol (CAP), ceftriaxone (CRO)	Zn-MOF	1,2-benzene- diacetic acid	goat serum	recovery: 92–104% for CAP 93.2–98% for CRO linear range: 5 × 10^−6^-2 × 10^−4^ mM for CAP 5 × 10^−6^–5 × 10^−5^ mM for CRO LOD: 12 ppb for CHL 3.9 ppb for CRO *K*_SV_: 2.23 × 10^6^ M^−1^ for CAP *K*_SV_: 9.82 × 10^6^ M^−1^ for CRO	energy absorption competition	[166]
Through a solvothermal reaction, a two-dimensional zinc(II)-based MOF was produced, and it demonstrated good chemical stability in the pH range of 2 to 12 as well as potent fluorescence with excitation and emission maxima of 270 and 290 nm, respectively, for the detection of antibiotics.						

chloramphenicol	MIP/UiO-66-NH_2_	2-amino- terephthalic acid	honey milk	recovery: 98–105% 96–105% linear range: 0.16–161.56 µg·L^−1^ LOD: 0.013 µg·L^−1^ *K*_ec_: 0.0272 × 10^6^ L·g^−1^	fluorescence enhancement	[167]
For the selective detection of chloramphenicol residues in milk and honey, a luminescent sensor based on nanostructured, molecularly imprinted polymer-coated zirconium-MOF was developed. As a result of the MOF’s strong fluorescence property paired with coated MIP’s particular binding sites for analyte identification, antibiotics could be detected selectively and sensitively.						

chloramphenicol	Cu/UiO-66@aptamer	fluorescent dye 6-carboxy-x- rhodamine (ROX)	CAP Eye drop	recovery: 96.45–103.9% linear range: 0.2–10 nmol/L LOD: 0.09 nmol/L	fluorescence enhancement	[168]
By coordinatively connecting the MOF nanomaterial UiO-66 with copper ions, bimetallic MOF (Cu/UiO-66) was synthesized. A phosphate and fluorescent dye double-labelled chloramphenicol aptamer was coated on the bimetallic MOF to improve the sensitivity and selectivity of the sensor. All single-stranded aptamer were adsorbed on the surface of Cu/UiO-66 in the absence of CAP, and their fluorescence was subsequently quenched by photoinduced electron transfer. The fluorescence was recovered when CAP was present because it combined with nucleic acid aptamers to generate a unique spatial structure.						

cefixime	Zn-MOF	3,5-di(1,2,4-triazol- 1-yl) pyridine (DTP)	—	linear range: 0–0.05 mM LOD: 2.60 × 10^−7^ M *K*_SV_: 2.94 × 10^4^ M^−1^	IFE, FRET	[47]
Here, two Zn-MOFs were synthesized using multiple ligands, the MOF containing 1,4-naphthalenedicarboxylic acid exhibited improved and better fluorescence quenching efficiencies.						

ciprofloxacin (CIP), norfloxacin (NFX)	TB-MOF	rhodamine B, 3-(3,5-dicarboxyl- phenyl)-5-(4- carboxylphenl)-1*H*-1,2,4-triazole (H_3_dcpcpt)	—	LOD: 716 ppb for CIP 201 ppb for NFX *K*_SV_: 1.67 × 10^4^ M^−1^ for CIP *K*_SV_: 5.71 × 10^4^ M^−1^ for NFX	PET, IFE	[169]
By using a solvothermal process, a lanthanide-MOF was synthesized. Rhodamine B (RhB) was then trapped in the Tb-MOF channels using an ion exchange procedure to create a high-performance fluorescence sensor. The RhB@Tbdcpcpt demonstrated stable columinescence of RhB and Tb^3+^ ions throughout the whole excitation range for the detection of different antibiotics.						

ornidazole (ONZ), metronidazole (MNZ), dimetridazole (DMZ)	Cd-MOF	3-(3,5-dicarboxyl- phenyl)-5-(4- carboxylphenyl)- 1*H*-1,2,4-triazole (H_3_DBPT)	—	LOD: 5.0 μM (1.10) ppm for ONZ 10.0 μM (1.71) ppm for MNZ 10.0 μM (1.41) ppm for DMZ *K*_SV_: 2.4 × 10^4^ M^−1^ for ONZ 2.0 × 10^4^ M^−1^ for MNZ 1.7 × 10^4^ M^−1^ for DMZ	PET, FRET	[170]
The multifunctional ligand was used to successfully bridge hexanuclear "Cd_6_" clusters, resulting in the creation of a chemically and thermally stable fluorescent Cd-based MOF with an open Lewis-basic triazolyl active site. With the use of the MOF material’s solvent-dependent fluorescent and ligand-based photoluminescence intensities, nitroaromatic antibiotics could be specifically detected in a variety of samples.						

cephalexin	ZIF-8	gCDs and AuNCs	milk	recovery: 97.2–106.3% linear range: 0.1–6 ng/mL LOD: 0.04 ng/mL		[171]
As a sensor for cephalexin detection, a ratiometric fluorescent probe based on in situ incorporation of green-emitting carbon dots (gCDs) and gold nanoclusters (AuNCs) into ZIF-8 (MOF) was developed. The fluorescence intensity of the gCDs remained constant while the fluorescence intensity of the AuNCs dropped in the presence of cephalexin. As a sensing signal, the ratio of fluorescence intensities at two clearly defined wavelengths was employed. There was some correlation between the ratio of luminescence intensity and cephalexin concentration.						

ascorbic acid	Zn-MOF-5	rhodamine blue dye and 2,5-dimethyl- terephthalic acid	rat brain microdialysates	linear range: 1–25 μm LOD: 0.31 μm	fluorescence enhancement	[172]
Through a simple one-pot synthesis procedure, Zn-MOF-5 was synthesized and fluorescent rhodamine blue dye was embedded. Through a "on-off-on" fluorescence response mechanism, the fabricated RhB@DiCH_3_MOF-5 was used for the simultaneous detection of ascorbic acid and Fe^3+^. When Fe^3+^ was added to the sensor solution, there was a noticeable fluorescence quenching. Ascorbic acid caused the reduction of Fe^3+^ to Fe^2+^. The quenched fluorescence of the probe could then be effectively recovered as a result. These techniques allowed for the MOF-regulated selective ascorbic acid detection in rat brain microdialysates.						

ascorbic acid	Eu-MOF-253-NH_2_	blue emission: H_2_N-BPDC^2−^ red emission: Eu^3+^	—	linear range: 1.6–100 μM LOD: 0.73 μM	fluorescence enhancement	[173]
This work describes the sequential mixed-ligand self-assembly and post-synthesis synthesis of a nanoscale dual-emission multivariate Eu-MOF-253-NH_2_ for ratiometric sensing of hypochlorite and ascorbic acid. Strong blue emission from ligands is sensitive to hypochlorite, but practically constant red emission from Eu^3+^ is sensitive to ascorbic acid.						

ascorbic acid	MOF-Cd-abtz	1-(4-aminobenzyl)-1,2,4-triazole (abtz)	urine human serum	recovery: 98–103% 93–99% linear range: 0.1–140 μM LOD: 75 nM *K*_SV_: 1.18 × 10^4^ L·mol^−1^	static quenching	[174]
As a fluorescence sensor, a Cd-based MOF was developed. The method used in this study is extremely selective, sensitive, accurate, and practical for rapidly and precisely detecting ascorbic acid in biological fluids without the need for laborious modification or immobilization of a fluorescent probe.						

folic acid	MOF-AgClO_4_-abtz	1-(4-aminobenzyl)-1,2,4-triazole (abtz)	human serum plasma serum	recovery: 95.3–104.6% 101.4–104.1% linear range: 0.1–30 μM LOD: 49 nM	fluorescence quenching, IFE	[175]
A highly sensitive and selective fluorescence sensor for the detection of folic acid in biological samples was developed using a water-stable Ag-based MOF. The MOF demonstrated high chemical stability, good water dispersibility, and a 78.97% fluorescent quantum yield, indicating significant application potential for fluorescence sensing.						

tetracycline (TC) and folic acid (FA)	Ag@Cu-MOF	NH_2_-H_2_BDC	—	linear range: 0.1–38 μM for TC 0.5–73 μM for FA LOD: 0.016 μM for TC 0.27 μM for FA	IFE, hydrogen bonding	[176]
For the detection of tetracycline and folic acid, bimetallic (Ag-Cu) MOF nanosheets were produced at room temperature for 4 h. The fluorescent sensor displays a potent emission peak and a steady fluorescence emission signal when fluorescence is excited. The fluorescence signal of the fluorescent probe is quenched by the addition of the target analyte TC. The fluorescence signal of the probe increased with the addition of FA. This enables the highly sensitive and precise dual-signal detection of tetracycline and folic acid.						

^a^IFE: inner filter effect; PET: photoinduced electron transfer; FRET: Förster resonance energy transfer.

**Table 4:** MOFs-based fluorescence sensor for detection of hormones in different samples.^a^

Analyte	Sensing materials	Ligand/reagent	Real sample	Analytical performance	Mechanism	Ref.

dopamine	Tb-MOF	*N*-carboxymethyl- (3,5-dicarboxyl)- pyridinium bromide	urine serum	recovery: 96.14–103.34% 98.68–104.32% LOD: 0.41 μM *K*_SV_: 59918 M^−1^	Competitive absorption of excitation and emission of Tb-MOF, static quenching.	[177]
Using a hydrothermal method, a three-dimensional Tb-based MOF was synthesized without the use of any further post-processing steps. For the label-free detection of dopamine, the MOF performs as a reliable fluorescence sensor. Due to a partial overlap between the MOF’s excitation spectrum and the polymerized dopamine’s absorption spectrum, the MOF’s green luminescence was quenched by the polymerized dopamine under the optimum conditions.						

melatonin	Zr-BDC@MIP	H_2_BDC	grape juice sour cherry juice cherry juice	recovery: 91.33–97.2% 98.06–98.96% 100.4–102% linear range: 1–100 ng/mL LOD: 0.18 ng/mL	fluorescence enhancement	[178]
The molecularly imprinted polymer (MOF@MIP) that contained Zr-MOF was used to produce the fluorescent probe. The unique recognition abilities of MIP and the fluorescence capabilities of the MOF, which considerably enhanced the selectivity and operation of the applied sensor, are combined to provide the sensor material its excellent performance.						

insulin	lanthanide-MOF	terphenyl-3,4″,5- tricarboxylic acid, rhodamine blue dye, aptamer	human serum	recovery: linear range: LOD: 0.0012 μM *K*_SV_: 2.99 × 10^5^ M^−1^	hydrogen bonding between insulin and FAM-P	[179]
Three-dimensional cluster-based MOFs made of rare-earth elements (Re = Dy, Gd, Ho, Pr, and Sm) were synthesized. Then, to develop the composite material RhB@MOF-Re with a high quantum yield, the fluorescent dye rhodamine B (RhB) was added to the MOF framework. The RhB@MOF-Gd based sensor was further modified with an aptamer and used as a superb bifunctional MOFs-based sensing platform to detect Al^3+^ and insulin with a low detection limit in the human serum solution.						

dopamine	CdI_2_–MOF	1-(4-aminobenzyl)- 1,2,4-triazole) (abtz)	urine	recovery: 94.5–102% linear range: 0.25–50 μM LOD: 57 nM	fluorescence enhancement	[180]
For label-free detection of dopamine, a Cd-based MOF was synthesized using the solvo-thermal synthesis approach and employed as a "off–on" fluorescent switch. Dopamine effectively recovered the fluorescence signal of CdI_2_-MOF in a "off–on" state after effectively quenching it with KMnO_4_.						

dopamine	Cu@Eu–MOF	1,3,5-benzene- tricarboxylic acid	human serum	recovery: 98.1–110.1% linear range: 0.04–30 μM LOD: 0.01 μM	fluorescence enhancement	[181]
Cu@Eu-MOF was developed by synthesizing a multifunctional lanthanide metal-MOF based on Eu^3+^ and then further modifying it with Cu^2+^. The doping of copper promoted the amplification of blue fluorescence and boosted the sensitivity of dopamine detection.						

17β-estradiol	Tb-MOF	Ru(bpy)_3_^2+^	lake water	recovery: 97.6–104% linear range: 50–1000 pM LOD: 50 pM	fluorescence enhancement	[182]
A high-performance MOF structure with the dual functions of a catalyst and fluorescent sensor for the degradation and detection of 17-estradiol and its derivatives was developed using a catalytic hemin, luminescent lanthanide ion (Tb^3+^), and bridging ligand, as well as an antenna molecule of Tb^3+^ fluorescence.						

insulin	UiO-67	Ru(bpy)_3_^2+^, Au nanoparticles and SiO_2_	human serum	recovery: 98–100% linear range: 0.0025–50 ng·mL^−1^ LOD: 0.001 ng·mL^−1^	resonance energy transfer	[183]
For the purpose of detecting insulin, a ruthenium(II) complex was added to the UiO-67 MOF and modified with gold nanoparticles. The combined characteristics of the sensor material’s pristine components are thought to be the cause of the detection mechanism. Due to the huge specific surface area and porosity of UiO-67, the loaded Ru(bpy)_3_^2+^ increased electrochemiluminescence efficiency.						

serotonin	Tb^3+^-NOTT-220	biphenyl-3,3',5,5'- tetracarboxylatic acid, Bi(NO_3_)_3_⋅5(H_2_O) and piperazine	serum	linear range: 0–200 μM LOD: 0.57 μM in water 1.21 μM in serum	Effects of dynamic quenching, competitive light energy absorption between the ligand and analyte, and energy transfer from the ligand to Tb^3+^.	[184]
By integrating Tb^3+^ into a metal-organic framework based on bismuth (Tb-NOTT-220), a ratiometric fluorescence sensor that can quickly and accurately detect serotonin in serum media was developed. When serotonin was exposed to the sensor, the ligand’s ability to recognize the target analyte was hindered, which was accompanied by an increase in the ligand’s fluorescence intensity and a decrease in the emission of Tb^3+^.						

serotonin	Eu-doped-UiO-66	*p*-phthalic acid and 2,3-pyridine- dicarboxylic acid	human serum	recovery: 99.51–107.69% linear range: 0.05–6.54 µM LOD: 0.013–0.15 µM *K*_SV_: 3.20 × 10^5^ M^−1^	IFE, PET, dynamic quenching process, coordination interactions between serotonin and Eu^3+^.	[185]
A water-stable fluorescence biosensor for the precise detection of neurotransmitters and their metabolites was created by synthesizing UiO-66 MOF using a hydrothermal method and then doping it with Eu^3+^. Smartphone-assisted RGB colour values for serotonin and its metabolite identification were created using the Eu-MOF-embedded test strip as a component.						

dopamine and glutathione (GSH)	UiO-66-NH_2_	2-aminoterephthalic acid	human serum	recovery: 97.3–102.8% for DA 94.3–100.3% for GSH linear range: 4–50 μM for DA 1–70 μM for GSH LOD: 0.68 μM for DA 0.57 μM for GSH	FRET	[186]
A hydrothermal approach was used to develop an UiO-66-NH_2_ MOF-based ratiometric fluorescence probe for the sensitive detection of dopamine and reduced glutathione. By observing the ratiometric fluorescence intensity, it was possible to simultaneously detect reduced glutathione and the dopamine copolymer that quenched the fluorescence of UiO-66-NH_2_ MOF.						

triiodothyronine (T3)	Cu-MOF-NPs	2,3-diamino-5- bromopyridine	serum	recovery: 99.53 and 102.0% linear range: 0.0–200.0 ng/dL LOD: 0.198 ng/dL	dynamic energy transfer between Cu-MOF and T3 hormone	[10]
Triiodothyronine was detected in biological fluids by a considerable quenching of the photoluminescence intensity using a copper metal-MOF that was developed using a simple method.						

diethylstilbestrol	Zr-MOF	*trans*-4,4′-stilbene- dicarboxylic acid: (H_2_sbdc)	milk fish extract	recovery: 99–102.1% 97.9–99% linear range: 0–10 µM LOD: 89 nm	PET	[187]
An enzyme-assisted MOF-fluorescent-sensing probe was created using a nanoscale MOF based on stilbene. Notably, diethylstilbestrol was indirectly detected through the quenching brought on by the result of its enzymatic oxidation, not by causing the fluorescence response of Zr-MOF.						

17β-estradiol	NKU-103(EuTb)	2,5-furan- dicarboxylic acid (H_2_FDA)	—	LOD: 2.7 × 10^6^ *K*_SV_ at 545 nm: 2.005 × 10^3^ M^−1^ *K*_SV_ at 616 nm: 4.501 × 10^3^ M^−1^	static quenching process and energy transfer from Tb^3+^ to Eu^3+^ ions in the EuTb-MOF	[188]
To identify 17-estradiol, a mixed-lanthanide MOF-based luminescent Eu-TB based sensor was developed. Bilanthanide MOFs, as opposed to monolanthanide MOFs, benefits from the dual-emission centres to efficiently minimize the systematic errors, such as those from the concentrations, excitation wavelength and slit width.						

^a^IFE: inner filter effect; PET: photoinduced electron transfer; FRET: Förster resonance energy transfer.

### Opto-electrochemical nanostructured sensors: practical challenges and future perspectives

The use of opto-electrochemical sensors in numerous environmental, agro-industrial, and biomedical applications has great potential. Despite the enormous success of glucose sensors, much more effort is still needed to develop opto-electrochemical sensors that are commercially feasible or market-ready. For instance, the COVID-19 pandemic has shown how crucial and urgent it is to have reliable, affordable, and rapid diagnostic sensing tools. Opto-electrochemical sensors have a number of advantages over other currently used sensing approaches or techniques. They can be mass-produced and are compact enough to fit into portable devices. The MOF opto-electrochemical sensors have shown a number of promising results, and in particular, they have produced good results when applied to real samples. Nevertheless, there are still obstacles preventing their commercialization and other challenges. The following is a brief discussion of some of these challenges, recent developments, and potential future advances.

It is important to note that while many authors have used opto-electrochemical sensors to detect analytes in actual samples, very few authors have evaluated the LOD and sensing capabilities of these sensors against more established techniques such as HPLC, AAS, and ELISA, to confirm their sensitivity and selectivity. Additionally, the majority of the published studies base their validation of the sensor performance solely on the real samples produced in the lab of the author without comparing those performances to those of actual samples produced by other researchers in other labs. As a result of the data being based solely on the author’s laboratory investigations, the commercialization of such sensors is constrained. Therefore, for the sake of practicality, findings from conventional analytical methods and actual samples from other labs under numerous different conditions should also be included when reporting the sensor performances.

The application of “actual samples” is arguably the most significant test to confirm the reliability of a sensor. It is impossible to certify a sensor as a trustworthy sensing tool if it is not stable or functioning in real samples. Numerous species that easily adsorb onto surfaces are frequently present in real samples. Since non-specific adsorption tends to dramatically reduce the repeatability, specificity, and sensitivity of the sensors, it has been one of the key obstacles to using electrochemical sensors in practical applications. With electrochemical sensors, sweat, blood serum, human serum, urine, blood serum, saliva, interstitial and tear fluids are the most often utilized real samples.

All of these practical samples are affected by the matrix effect, which tends to negatively obstruct the detection of a particular analyte and reduce recovery values and sensor sensitivity [189–192]. For instance, saliva samples must frequently be filtered or diluted in order to be utilized as actual samples because they are complicated mixtures. The biggest issue with urine samples is the variety of pH ranges that can affect the position of peak potential and the height of current intensity, and create stability problems [191]. Tear fluid has also recently attracted a lot of attention due to its reduced complexity and ease of access for non-invasive sampling procedures, however the pH value can fluctuate, the sample volume is small, and the composition of tears shed in response to emotion and irritation may differ.

Researchers routinely dilute real samples to lower the interference effect below a tolerable threshold, which aids in overcoming the matrix effect. However, the sample is further from reality the more diluted it gets. Without sample dilution or processing, sensors should ideally be able to perform well with pure actual samples such as whole blood [189]. Researchers are addressing this issue when it comes to point-of-care sensing platforms and diagnostic sensors with cutting-edge materials and techniques to enhance sensor performance when utilized with actual samples. In particular, the development of non-charged and hydrophilic layers has been employed to obstruct matrix adsorption on the surface of the electrochemical sensors using both active and passive approaches. Lichtenberg et al. provided a comprehensive review of these techniques [192]. Although molecularly imprinted polymers have been used to modify electro-active sensor materials such as MOFs to increase the specificity and selectivity of the target analytes for antibiotics and hormones, future research should also concentrate on the development of simple-to-prepare reagents capable of suppressing the matrix effects in actual samples.

A fluorescence labelling procedure is initially needed for fluorescence-based nanostructured sensors in order to detect analytes such as antibiotics and hormones that are not fluorescent by nature. Linking the fluorescent tags to the biomolecules may require additional sample preparation steps. To avoid additional sample preparation stages, some studies have concentrated on the integration of optical sensing platforms with microfluidic channels that contain sample filtration/separation, target labelling, mixing, and washing. Prefabricating nanostructured materials with recognition moieties for particular analyte detection is another strategy. For instance, MOFs with strong fluorescence properties can be modified using aptamers or molecularly imprinted polymers in disposable, low-cost materials such as filter paper. This method can greatly cut down on time-consuming procedures and improve the system’s effectiveness.

The operation of the potentiostat for an electrochemical sensor still requires external instruments. A laptop or PC, for instance, is frequently needed to operate the potentiostat or process the data. This is a challenge, particularly when deploying the electrochemical sensor for routine analysis in the field. Single-board computers, such as Raspberry Pi, Arduino, and smartphones, can be used as a practical solution to tackle challenging tasks simultaneously, such as data analysis, image processing, and controlling peripheral devices. Additionally, by enabling real-time analyte detection for numerous other applications, including healthcare and environmental monitoring, the portable electrochemical sensor device will perform better when connected to the Internet of Things.

Obtaining a low limit of detection is another key problem that needs to be addressed for the development of electrochemical sensors that are commercially viable or market-ready. When developing a commercially viable electrochemical sensor, the LOD is a crucial parameter. This metric represents the smallest concentration or amount of a particular analyte that may be accurately identified while maintaining a respectable signal-to-noise ratio. Because analytes are frequently present in real samples only in trace amounts, developing a sensor with a low LOD is essential. LODs with values as low as picomole and femtomole levels have been reached in the case of some ultra-sensitive electrochemical sensors due to the development of nanomaterial-modified surfaces.

Researchers have developed many nanostructured materials to modify the electrodes. Since drop casting techniques are frequently not as reproducible as one would want, it is still difficult to modify electrodes with nanostructured materials based on these techniques. The conformation and topology of these nanomaterials, for instance, may change between each sensor since it is challenging to manage the immobilization of nanoparticles with varied populations of size and shape when making large quantities of sensor electrodes. Since it is not practical to evaluate each sensor made in mass-production facilities, sensor-to-sensor repeatability is crucial during the manufacturing process. Drop casting frequently results in uneven coatings of different thicknesses. Although spraying/spray coating creates uniform coatings with the ability to vary thickness, the majority of commonly used spin/spray coating devices are still expensive and not made for the electrodes used in electrochemical sensors that have smaller surfaces, such as disposable screen-printed electrodes. Future research should focus on developing simpler, more affordable mechanized immobilization and coating systems that can be used to modify commercially viable electrodes with uniform and consistent nanostructured surfaces.

Notably, the stability of sensors has also proven challenging, restricting the use of these devices under extreme conditions and in remote places. As a result, it is essential to develop sensors that can function for a considerable amount of time. Sensors are frequently distinguished by their shelf-life. Due to problems with aggregation and flaking of layers modified by nanoparticles, long-term stability may become a significant concern when utilizing nanomaterials. It is anticipated that the high surface area and stability of MOFs as hybrid electrode materials will enable the production of remarkable sensors with superior stability and minimal loss in analytical performance by combining the excellent electrical conductivity of inorganic nanomaterials with the analyte recognition capabilities of molecularly imprinted polymers and aptamers.

Poor electrical conductivity and low analyte specificity are the main drawbacks of conventional MOF-based electrochemical sensors. Hybrids and the development of nanocomposite materials based on MOFs are recent advancements to deal with these problems. For instance, scientists are developing multimetallic MOFs. Some newly developed bimetallic MOFs have been used gradually in the electrochemical sensor industry because they offer electroanalytical capabilities superior to those of conventional MOFs. Jalal et al. [193] showed that bimetallic MOFs exhibit greater electrochemical activity than monometallic MOFs for electrochemical detection of the antibiotic doxorubicin because of the synergistic interaction between the metal centres and the electrochemically active ligands.

An additional strategy is the development of MOF-based nanocarriers containing molecular recognition components (such as proteins, enzymes, and antibodies) and signals amplification components (metal nanoparticles or enzymes). The great porosity of MOFs makes it possible for them to hold a lot of active molecules, and the numerous surface functional groups open up the possibility of biomolecular modification via hydrogen bonding, π–π stacking, covalent bonding, and other intermolecular interactions. Excellent specificity, precision, and stability are provided for the detection of analytes on the complex matrix by the addition of specialized recognition components. Additionally, the incorporation of bimetallic nanoparticles in MOF structures could boost the efficiency of electron transfer at the electrode interface in addition to offering numerous catalytic sites.

Notably, the challenges with opto-electrochemical sensors described above apply not only to the detection of hormones or antibiotics but also to other applications in agriculture, forensic science, food safety, environmental monitoring, and defence and military applications. Fortunately, the discovery made possible by the use of diverse nanostructured materials constitutes a substantial advancement with implications for all the sectors indicated above. More research should concentrate on developing market-ready products using lab-developed sensors that have undergone extensive field testing and stability investigations over a period of many months.

## Conclusion

For the development of reliable high-performance sensors for the monitoring and detection of trace analytes such as pharmaceuticals and their metabolites in complex matrices, high sensitivity and low detection limits are in fact the essential requirements. The focus of this review is on opto-electrochemical sensing materials based on functional metal-organic framework nanostructured materials for the detection of various pharmaceuticals, in particular, antibiotics and hormones, in complex matrices. Numerous opto-electrochemical sensing applications are made possible by the high porosity, tunable topology, high quantum yield, fluorescence capabilities, and ease of functionalization of MOFs. Their distinctive characteristics, including electrical, optical, and chemical properties, are also reviewed and critically discussed. Numerous optical sensing systems are reviewed and discussed, with a focus on those connected to fluorescence sensing and electrochemical processes.

The challenges with opto-electrochemical sensors based on MOFs are highlighted. Also mentioned are recent developments that aim to ease these technical difficulties. In this regard, it is demonstrated that the recognition potential, electrocatalytic, fluorescence emission, and analytical performance of MOF sensors can all be enhanced by the incorporation of synthetic or improved recognition elements such as molecularly imprinted polymers, aptamers, and mixed nanoparticles. Summaries of previously published opto-electrochemical sensors based on MOFS and their analytical parameters are provided in Tables 1–4. We critically evaluate and discuss key findings on mechanisms, synthesis approaches, actual samples used, and ligands and reagents for the synthesis of the MOFs.

It should be noted that MOFs and their derivatives are not just useful for detecting hormones and antibiotics; rather, their inherent properties have shown that they can be applied to a variety of fields, including healthcare (point-of-care), the environment, food quality and safety, and agriculture. Also, they have a very promising future in the practical detection of explosives. It is envisaged that future research will combine MOFs with other innovative nanostructured materials, such as Mxenes, transition metal chalcogenides, quantum dots, and artificial nanozymes, to develop hybrid materials with synergistically enhanced characteristics and stability.
